# Differences in antimicrobial resistance between *exoU* and *exoS* isolates of *Pseudomonas aeruginosa*

**DOI:** 10.1007/s10096-025-05132-6

**Published:** 2025-04-22

**Authors:** Tanzina Akter, Fiona Stapleton, Mark Willcox

**Affiliations:** 1 School of Optometry and Vision Science, Faculty of Medicine and Health, University of New South Wales (UNSW), Sydney, NSW 2052 Australia; 2 Microbial Biotechnology Division, National Institute of Biotechnology (NIB), Dhaka, 1349 Bangladesh

**Keywords:** *ExoU*, *ExoS*, *Pseudomonas aeruginosa*, Antibiotics resistance, DNA gyrase and topoisomerase IV, Efflux pumps, Mutations

## Abstract

**Purposes:**

This study compared antimicrobial resistance between *exoU* and *exoS Pseudomonas aeruginosa* strains isolated from microbial keratitis (MK) and examined their resistance genotypes.

**Methods:**

The presence of *exoU* and *exoS* was determined in 187 MK isolates using PCR. Minimum inhibitory concentrations of ciprofloxacin, levofloxacin, gentamicin, and tobramycin were measured. Whole genome sequencing of 39 isolates was used to identify resistance genes via Resfinder. Mutations in key genes, including DNA gyrase, topoisomerase IV, efflux pumps, and DNA repair systems, were analyzed using Geneious Prime. Functional effects of novel SNPs were predicted using SIFT.

**Results:**

Antibiotic resistance was significantly higher in *exoU* than *exoS*: 38.2% vs. 20.5% for ciprofloxacin, 29.1% vs. 12.1% for levofloxacin, 40% vs. 23.5% for gentamicin, and 29.1% vs. 14.4% for tobramycin (all *p* < 0.05). *ExoU* isolates exclusively had mutations in GyrA (Thr83Ile) and ParC (Ser87Ile), as well as in efflux pump regulators MexZ (Gly89Ser), NalC (Asp79Glu) and MexS (Val73Ala) (*p* < 0.01). They also more frequently harbored the acquired resistance genes *aph(6)-Id* (55% vs. 0%) and *aph(3’’)-Ib* (60% vs. 5.3%) and had higher mutation rates in DNA repair genes *mutL* (70% vs. 15.8%) and *mutS* (45% vs. 5.3%) (*p* < 0.01). Mutations in *gyrA*, *parC*, efflux pump (*mexB*,* mexD*,* mexY*) and regulator (*mexZ*,* nalC*,* mexS*) genes correlated with fluoroquinolone resistance (*R* ≥ 0.33; *p* ≤ 0.04). Possession of *aph(3’’)-Ib*, *aph(6)- Id* and SNPs in efflux pump regulators *mexZ* and *parR* were associated with aminoglycoside resistance.

**Conclusion:**

*ExoU* strains exhibited more resistance genes and mutations, contributing to higher resistance to fluoroquinolones and aminoglycosides.

**Supplementary Information:**

The online version contains supplementary material available at 10.1007/s10096-025-05132-6.

## Introduction

Microbial keratitis (MK) is a severe sight-threatening ocular infection of the cornea, a transparent structure located at the front of the eye, covering both the pupil and iris. MK affects millions of individuals across the world annually [1]. *Pseudomonas aeruginosa* is a leading cause of MK [2], and responsible for ≥ 66% of cases of MK associated with wearing contact lenses [3, 4, 5]. *P. aeruginosa* keratitis is characterized by ocular pain, infiltration of inflammatory cells, stromal destruction, and if inappropriately treated, corneal perforation within 48–96 h of infection, with potential vision loss [6].

*P. aeruginosa* isolates can be classified into two lineages, differentiated by the possession of the genes *exoU* and *exoS*, which are usually mutually exclusive [6, 7]. The products of both of these genes are exported via the type III secretion apparatus of *P. aeruginosa* [6, 7, 8]. The *exoU* gene encodes for a phospholipase that is associated with acute cytotoxicity of mammalian cells [9, 10]. The *exoS* gene encodes a bifunctional protein with a GTPase activating domain and an ADP ribosyltransferase domain, that is associated with invasion of strains into mammalian cells and the eventual death of host cells through apoptosis [11, 12, 13, 14]. These factors are known to play important roles in the pathogenesis of MK [10, 15]. *ExoS P. aeruginosa* strains are generally more common among keratitis isolates, while *exoU* strains, though less frequent [10, 16, 17], are associated with more severe disease outcomes due to their potent cytotoxic effects than the *exoS* [18, 19].

Due to its rapid progression, *P. aeruginosa* keratitis is typically managed empirically with initial treatment using topical antibiotics such as ciprofloxacin or other fluoroquinolones, or with an aminoglycoside (such as gentamicin) fortified with cephalosporin (such as ceftazidime) [16, 20, 21]. However, *P. aeruginosa* exhibits varying resistance patterns to antibiotics across different regions. In Iraq, China, and India resistance to ciprofloxacin ranges from 24 to 56% of isolates, whereas in Australia resistance is low at around 5% of isolates [21, 22, 23]. There is a significant correlation between the presence of *exoU* and resistance to fluoroquinolones in *P. aeruginosa* [24, 25], and evidence of co-selection between the *exoU* and antimicrobial resistance, including resistance to disinfectants, in both ocular and non-ocular *exoU* strains [19, 25, 26, 27]. This raises concerns about the potential role of antibiotic resistance in the evolution of more virulent *P. aeruginosa* strains.

Resistance to fluoroquinolones may occur through three main mechanisms. Firstly, acquisition of chromosomal mutations in the quinolone resistance-determining region (QRDR) of genes *gyrA* and *gyrB* that encode the GyrA and GyrB subunits of DNA gyrase and *parC* and *parE* genes that encode the ParC and ParE subunits of topoisomerase IV [28]. Secondly, by overproducing efflux pumps or altering cell permeability to reduce the intracellular concentration of fluoroquinolones. Four efflux pumps, MexAB-OprM, MexCD-OprJ, MexEF-OprN, and MexXY-OprM, are involved in expelling fluoroquinolones, and their overexpression is linked to mutations in their regulatory genes *mexR*,* nalC*,* nalD*,* nfxB*,* mexT*,* mexS* and *mexZ* [29]. Finally, *P. aeruginosa* can obtain transferable quinolone resistance [30, 31, 32], such as the *crpP* gene that encodes a ciprofloxacin-modifying enzyme or the *qnrVC1*gene [29].

In case of aminoglycosides, *P. aeruginosa* may develop resistance due to enzymatic modification of the active chemical groups in the antibiotics by aminoglycoside-modifying enzymes (AMEs) such as aminoglycoside phosphoryltransferase (APH), aminoglycoside nucleotidyltransferase (ANT), aminoglycoside acetyltransferase (AAC), and aminoglycoside adenylyl transferase (AAD) [33]. All *P. aeruginosa* strains possess *aph(3’)-IIb*, whilst the others can be acquired through various mechanisms [34, 35, 36]. In addition, aminoglycoside resistance is also linked to reduced cell permeability, increased drug efflux by the MexXY-OprM pump due to mutations in key repressors genes including *mexZ* [37], the aminoglycoside-inducible anti-repressor *armZ* [38, 39], and the two component system *parRS* [40], as well as mutation in *fusA1*, which encodes the elongation factor EF-G1A [41], and ribosomal changes induced by 16 S rRNA methylase [36, 40].

The aim of the current study was to compare the antimicrobial resistance of *exoU* and *exoS* strains to fluoroquinolones and aminoglycosides that commonly prescribed for keratitis, and to study the genetic variations between *exoU* and *exoS* keratitis isolates that were associated with phenotypic resistance.

## Materials and methods

### *P. aeruginosa* isolates

One hundred and eighty-seven *P. aeruginosa* keratitis isolates from Australia (*n* = 132) and India (*n* = 55) (collected from 1993 to 2022) were used in this study. All the isolates were collected from different patients without identifiable patient data. These strains were stored at -80 °C in the microbial culture collection of the School of Optometry and Vision Science, UNSW Sydney, Australia and revived on nutrient agar (Oxoid Ltd., Basingstoke, Hampshire, UK). The VITEK-2 for gram-negative bacteria (BioMérieux, Baulkham Hills, NSW, Australia) was used to confirm the genus and species of bacteria according to the manufacturer’s instructions.

### DNA extraction and detection of *exoU* and *exos* genes of *P. aeruginosa*

Bacteria were grown on Tryptone Soy Broth (TSB; Oxoid Ltd., Basingstoke, UK) overnight at 37^o^C and DNA was extracted from fresh cultures using the DNeasy Blood and Tissue Kit (Qiagen, Hilden, Germany) following the manufacturer’s instructions. The quantity and purity (the ratio of absorbance at 260 and 280 nm) of the extracted DNA was assessed using a spectrophotometer (Nanodrop ND-1000, Thermo Fisher Scientific, Waltham, MA). PCR was conducted to detect the presence of *exoU* and *exoS* genes using specific primers and cycling conditions described previously [42, 43] (Supplementary Tables 1 and 2). After PCR, amplified DNA fragments were visualized on 1.0% agarose gel containing Sybr Safe (Thermo Fisher Scientific, USA) to confirm the size of the fragments, and therefore the presence of the genes.

### Antibiotic susceptibility testing

The fluoroquinolones ciprofloxacin and levofloxacin (Sigma-Aldrich, St. Louis, Missouri, USA), and aminoglycosides gentamicin (Sigma-Aldrich) and tobramycin (Cayman Chemical Company, Ann Arbor, Michigan, USA) were selected for study.

Each antibiotic was serially diluted in cation-adjusted Mueller-Hinton broth (MHB; Becton Dickinson and Company, Franklin Lakes, NJ, USA) in sterile 96-well plates with concentrations ranging from 5120 µg/mL to 0.25 µg/mL. Bacteria, cultured overnight in MHB at 37 °C, were diluted with fresh MHB to a concentration of 1 × 10^5^ CFU/mL and 100 uL of diluted antibiotic and 100 uL of bacteria were added to the wells, followed by incubation at 37 °C for 18–24 h. The MIC was defined as the lowest antibiotic concentration that inhibited bacterial growth, indicated by no turbidity when measured spectrophotometrically at 660 nm. Susceptibility levels were interpreted using breakpoints (µg/mL, ciprofloxacin ≤ 1, 2, ≥ 4, levofloxacin ≤ 2, 4, ≥ 8, gentamicin ≤ 4, 8, ≥ 16, and tobramycin ≤ 4, 8, ≥ 16), from the Clinical and Laboratory Standards Institute (CLSI) and the European Committee on Antimicrobial Susceptibility Testing (EUCAST). Strains showing intermediate resistance and full resistance to antibiotics were categorized as resistant.

### Analysis of resistance genes and SNPs

From the 187 keratitis isolates, the whole genome sequences (WGS) of a subset of 39 isolates (20 *exoU*, 13 from India and 7 from Australia and 19 *exoS*, 6 from India and 13 from Australia) were available from NCBI (Bio project numbers: PRJNA590804 and PRJNA431326) [44, 45]. Among these 39 strains, some strains were resistant to one or multiple antibiotics and some were sensitive to all the tested antibiotics [44, 45]. These strains were examined for the possession of horizontally acquired antibiotic resistance genes for fluoroquinolones and aminoglycosides using the Resfinder database version 4.1.1 [46, 47]. The WGS data were also utilized in BLAST searches for the presence of *gyrA*, *gyrB*, *parC*, *parE*, *fusA1* and efflux pump genes *mexAB-oprM*, *mexCD-oprJ*, *mexEF-oprN*, *mexXY-oprM*, and the regulatory genes of the efflux pumps *mexR*, *nalC*, *nalD*, *nfxB*, *mexT*, *mexS*, *mexZ*, *amrZ*, *parR*, and *parS*. These gene sequences were aligned with the reference sequences of *P. aeruginosa* PAO1 (Accession number NC_002516.2) and *P. aeruginosa* PA14 (Accession number NC_008463.1) to identify SNPs associated with resistance to fluoroquinolones and aminoglycosides from *exoS* and *exoU* strains, respectively, using the Geneious prime tool version 2024.0.7 [48]. Similarly, SNPs in the DNA mismatch repair system genes (*mutL*, *mutS* and *uvrD*) were identified. Any mutations were further evaluated for their potential impact on protein function using the SIFT (Sorting Intolerant from Tolerant) algorithm, which predicts whether an amino acid substitution affects protein function based on sequence homology and the physical properties of amino acids [49]. Substitutions with a SIFT score ≤ 0.05 were considered functional mutations, indicating a potential disruption to protein function, while those with a score > 0.05 were considered tolerated variants, suggesting that the substitution was less likely to impair the protein’s functionality.

### Statistical analysis

Differences in the frequency of antibiotic susceptibility, acquired resistance genes and possession of SNPs between *exoU* and *exoS* groups were compared using Chi Squared test (GraphPad prism, 2024, v8.0.2.263). Correlations were examined using Spearman’s Rho. A p-value of < 0.05 was considered statistically significant for all analyses.

## Results

### Prevalence of *exoU* and *exos* genes and susceptibility to antibiotics

Based on the PCR results, 71% (132/187) of isolates possessed the *exoS* gene, while 29% (55/187) had the *exoU* gene (Supplementary Table 3). In the strains chosen for study, the prevalence of *exoS* in Australia was 74.2% (98/132) compared to 61.8% (34/55) in India, while *exoU* was more prevalent in India with 38.2% (21/55) compared to 25.8% (34/132) in Australia although the differences were not statistically significant (*p* = 0.11). For ciprofloxacin, the highest resistance was observed in *exoU* isolates PA210 and PA219 with MICs of ≥ 5120 µg/mL followed by PA221 (2560 µg/mL) and PA198 (1280 µg/mL; Supplementary Table 3). For levofloxacin, PA210 had the highest MIC (≥ 5120 µg/mL) then PA221 (2560 µg/mL) and PA219 (640 µg/mL). In the *exoS* group, PA75 had the highest MIC 512 µg/mL for ciprofloxacin and PA61 and PA227 (64 µg/mL; Supplementary Table 3) for levofloxacin.

For the aminoglycosides, *exoU* isolates PA31 and PA219 had the highest MIC value (≥ 5120 µg/mL) for gentamicin and PA33 showed the highest for tobramycin (≥ 5120 µg/mL). While in the *exoS* group, PA69 had the highest MIC value 1024 µg/mL for gentamicin and for tobramycin, PA56, PA65 and PA69 had MICs of 256 µg/mL. Resistance to ciprofloxacin (38.2% vs. 20.5%), levofloxacin (29.1% vs. 12.1%), gentamicin (40% vs. 23.5%), and tobramycin (29.1% vs. 14.4%) were significantly higher (*p* < 0.05) in the *exoU* group than the *exoS* group (Table 1).

**Table 1:** Antimicrobial susceptibility of 55 *ExoU* and 132 *exos* keratitis isolates

Antibiotics	Keratitis isolates = 187				
	*exoU* (55)		*exoS* (132)		P value
	S (%)	R (%)	S (%)	R (%)	
Ciprofloxacin	61.8	38.2	79.5	20.5	< 0.05
Levofloxacin	70.9	29.1	87.9	12.1	< 0.01
Gentamicin	60.0	40.0	76.5	23.5	< 0.05
Tobramycin	70.9	29.1	85.6	14.4	< 0.05

S = Susceptible; R = Resistant

Comparative analysis of phenotypic antibiotic susceptibilities of the available 39 MK WGS strains (20 *exoU* and 19 *exoS*), a subset of the 187 isolates, from NCBI (Bio project numbers: PRJNA590804 and PRJNA431326) was performed (Supplementary Table 4). For this subset of isolates, ciprofloxacin resistance (75% vs. 58%) was not statistically significantly different between *exoU* and *exoS* isolates (*p* = 0.32). Whereas *exoU* isolates were significantly more likely to be resistant (*p* < 0.01) to levofloxacin (55% vs. 11%). Among the aminoglycosides, 55% of *exoU* isolates were resistant to gentamicin, while no isolates from *exoS* group were resistant to gentamicin (*p* < 0.01). Tobramycin resistance was significantly more common (*p* < 0.01) in *exoU* (55% vs. 11%).

### Association of fluroquinolone resistance with mutations in target genes of QRDRs, efflux pumps and their regulatory genes and acquired resistance genes

The amino acid changes in the quinolone resistance-determining regions (QRDRs), efflux pumps (MexAB-OprM, MexCD-OprJ, MexEF-OprN, MexXY-OprM) and their regulatory proteins and possession of *crpP* and *qnrVC1* of 20 *exoU* and 19 *exoS* are presented in Supplementary Tables 5 and 6. Association of amino acid changes and acquired resistance genes of these strains with ciprofloxacin and levofloxacin resistance are presented in Table 2.

Mutations leading to amino acid changes in GyrA (Thr83Ile), ParC (Ser87Ile), NalC (Asp79Glu), MexS (Val73Ala), MexZ (Gly89Ser), MexB (Gly957Asp, Ser1041Glu, Val1042Ala), MexC (Glu251Gln, Ala262Glu, Ala277Thr), MexE (Ser8Phe), MexY (Ile536Val) were detected exclusively in *exoU* strains (*p* < 0.01; Table 2). Where present, *qnrVC1* was only found in *exoU* isolates (*p* = 0.11; Table 2). The possession of ParE Asp533Glu, MexR Val126Glu and OprN Ser13Pro were significantly more frequent in *exoU* strains (85%) than in *exoS* strains (21%, 26% and 37% respectively, *p* < 0.01). Similarly, MexX Ala30Thr had higher frequency in *exoU* strains (75%) compared to *exoS* isolates (5%). All strains had Gly71Glu NalC, deletion of AAs 81 and 82 followed by frameshift in MexT, and Asp249Asn in MexS. No strain had mutations in the GyrB and only PA157, a susceptible *exoS* strain, showed a mutation in the NfxB (Arg82Leu). *ExoS* strains more commonly (*p* ≤ 0.04) possessed the amino acid changes Asp68Gly and Met69Val in OprJ (Table 2). The SNPs that resulted in amino acid changes but that were not significantly different between *exoU* and *exoS* isolates are given in Supplementary Table 7.

**Table 2:** The significantly different amino acid changes resulting from differences in DNA sequences of DNA gyrase, DNA topoisomerase, efflux pumps and their regulatory genes, possession of acquired resistance genes between 20 *ExoU* and 19 *exos* keratitis isolates, and correlations with resistance to fluoroquinolones

Gene	Amino acid change	Frequency of possession (%)– exoU: exoS	Difference between exoU and exoS (*p*-value)	Correlation between amino acid change and resistance to ciprofloxacin (Spearman’s correlation coefficient *R*; *p*-value)	Correlation between amino acid change and resistance to levofloxacin (Spearman’s correlation coefficient *R*; *p*-value)
*gyrA*	Thr83Ile	65:0	< 0.01	0.50; < 0.01	0.77; < 0.01
*parC*	Ser87Ile	65:0	< 0.01	0.50; < 0.01	0.77; < 0.01
*parE*	Asp533Glu	85:21	< 0.01	0.40; 0.01	*0.30; 0.06*
*mexR*	Val126Glu	85:26	0.01	0.40; < 0.01	0.37; < 0.01
*nalC*	Asp79Glu	65:0	< 0.01	0.50; < 0.01	0.60; < 0.01
*mexS*	Val73Ala	35:0	< 0.01	0.33; 0.04	0.63; < 0.01
*mexZ*	**Gly89Ser**	35:0	< 0.01	0.33; 0.04	0.63; < 0.01
*mexB*	Gly957Asp	35:0	< 0.01	0.33; 0.04	0.63; < 0.01
	Ser1041Glu	55:0	< 0.01	0.44; < 0.01	0.68; < 0.01
	Val1042Ala	55:0	< 0.01	0.44; < 0.01	0.68; < 0.01
*mexC*	Glu251Gln	35:0	< 0.01	0.33; 0.04	0.63; < 0.01
	**Ala262Glu**	45:0	< 0.01	0.36; 0.03	0.68; < 0.01
	**Ala277Thr**	35:0	< 0.01	0.33; 0.04	0.63; < 0.01
*mexD*	Thr87Ser	35:5	0.02	0.34; 0.03	0.55; < 0.01
*oprJ*	Asp68Gly	10:37	0.04	-0.39; 0.02	-0.41; 0.01
	Met69Val	15:68	< 0.01	-0.41; 0.01	-0.41; 0.01
*mexE*	Ser8Phe	35:0	< 0.01	*0.05; 0.78*	*0.07; 0.68*
*oprN*	**Ser13Pro**	85:37	< 0.01	0.34; 0.04	0.37; 0.02
*mexX*	Ala30Thr	75:5	< 0.01	0.37; 0.02	0.57; < 0.01
*mexY*	Ile536Val	70:0	< 0.01	0.42; < 0.01	0.44; < 0.01
*crpP*	-	*65:42*	*0.11*	*0.11; 0.5*	*0.31; 0.06*
*qnrVC1*	-	*20:0*	*0.11*	*0.24; 0.14*	0.43; < 0.01

*Italicised* numbers indicate *p* > 0.05. Bold fonts indicate functional SNPs

*ExoU* ciprofloxacin resistant strains having amino acid changes in GyrA (Thr83Ile) and ParC (Ser87Ile) as well as novel functional amino acid changes in MexZ (Gly89Ser or Val43Gly) except in strains PA217 and PA82 showed elevated MICs (32 µg/mL to ≥ 5120 µg/mL) (Supplementary Table 5). In the case of PA217, along with GyrA Thr83Ile and ParC Ser87Ile, the elevated MIC to ciprofloxacin was associated with novel amino acid changes Ser5Pro and Gly213Asp in NalC and a frameshift in amino acids 160 to 213 in NalD, which were only found in this strain. PA82, along with GyrA Thr83Ile and ParC Ser87Ile, was the only strain to contain MexS Glu286Ala. Strains PA34, PA169, PA220 and PA233 had MICs to ciprofloxacin ranging from 2 to 8 µg/mL (Supplementary Table 5). These did not possess the additional amino acid changes outlined above, and only PA220 and PA34 possessed GyrA Thr83Ile and ParC Ser87Ile. PA34 exclusively possessed MexR Ala110Thr. PA34, PA169 and PA220 possessed NalC Asp79Glu (which was exclusive to *exoU* strains; Table 2). All levofloxacin resistant *exoU* strains had Thr83Ile and Ser87Ile mutations in GyrA and ParC and MICs ranging from 32 µg/mL to 2560 µg/mL, except for PA82 with a MIC of 4 µg/mL (Supplementary Table 5), this strain exclusively having MexS Glu286Ala. In PA202 and PA221 *exoU* resistant strains, the ParC protein had acquired a new functional mutation Ala599Val in addition to the Ser87Ile mutation.

Three novel functional mutations, leading to the amino acid changes Ala659Tyr, Ala918Gly, and Asn921Glu in GyrA were found in the *exoS* strain PA224, which was resistant to ciprofloxacin and another novel functional mutation leading to Asp652Tyr was carried by PA225 and PA227 which were resistant to both ciprofloxacin and levofloxacin. The amino acid changes Glu513Asp (new mutation) and Asp754Asn in ParC were observed only in PA40 and PA216 *exoS* strains, which had MIC values for ciprofloxacin of 4 and 64 µg/mL, respectively (Supplementary Table 5).

All the amino acid changes that were significantly different between *exoU* and *exoS* were also significantly correlated with resistance to ciprofloxacin (*R* ≤ 0.33; *p* ≤ 0.04), except for MexE Ser8Phe which was not related to ciprofloxacin or levofloxacin resistance and ParE Asp533Glu which was not correlated with levofloxacin resistance. Possession of *oprJ* SNPs leading to the amino acid changes Asp68Gly and Met69Val was negatively correlated with resistance to both fluoroquinolones. Possession of *crpP* was not associated with resistance to ciprofloxacin and levofloxacin, whereas possession of *qnrVC1* was associated only with resistance to levofloxacin (Table 2).

### Association between aminoglycosides resistance and mutations in efflux pumps, their regulatory genes and acquired resistance genes

Supplementary Table 8 shows the resistance profiles of 20 *exoU* and 19 *exoS* keratitis strains to gentamicin and tobramycin, amino acid changes resulting from SNPs in *armZ*,* parR* and *parS* and the various aminoglycoside-associated acquired resistance genes identified in these strains. SNPs in other associated genes (*mexX*, *mexY*, *oprM* and *mexZ*) in these isolates are provided in Supplementary Tables 5 and 6. The gene *aph(3’)-IIb* was present in all isolates of both lineages. The acquired resistance genes *aph(3’’)-Ib* and *aph(6)-Id* were more common in *exoU* and were correlated strongly with resistance to gentamicin and tobramycin (Table 3). The other aminoglycoside resistance genes (*aph(3’)-Ib*, *aph(3’)-VI*, *aac(3)-IId*, *aac(6’)-Ib3*, *aadA1*, *aadA10*, *aadA24*, *rmtD2*, and *rmtB*) were either rare or absent in both groups (Supplementary Table 8). None of the strains possessed mutations leading to amino acid changes in FusA and the SNPs leading to amino acid changes in ParS (Ala115Glu, Arg243His, Ser277Asn, Ala285Val, Gly388Asp) were not significantly different or correlated with aminoglycoside resistance. There were no significant differences or correlations in amino acid changes in ArmZ (Ala87Val, Leu88Pro, Asp104Glu, Gly157Asp, Asp207Asn, Asn238Ser, Val243Ala, Gly265Ser, Glu307Asp, Gly319Val, Ile346Val, Ala352Val), MexX (Leu12Pro, Thr15Ala, Asp25Glu, Lys26Glu, Pro28Ala, Glu29Asp, Glu31Gly, Ala33Thr, Asp35Glu, Lys46Arg, Val133Ile, Ser153Ala, Arg161Lys, Gln236Lys, Val331Leu, His338Arg, Gly344Asp, Gly384Asp, Val385Ala), MexY (Val240Met, Ala254Gly, Phe509Ser, Thr543Ala, Gly1035Asp, Ile1040Thr), MexZ (Asp83Glu, Leu138Arg, Leu196Ile), OprM (Val72Leu, Lys480Asn), ParR (Met59Ile, Ile93Thr, Thr135Ala, Gly232Asp), and ParS (Ala115Glu, Arg243His, Ser277Asn, Ala285Val, Gly388Asp).

**Table 3:** The significantly different amino acid changes resulting from differences in DNA sequences of efflux pumps and their regulatory genes, and acquired resistance genes of 20 *ExoU* and 19 *exos* keratitis isolates

Gene	Amino acid changes	Frequency of possession (%)– exoU: exoS	Difference between exoU and exoS (*p*-value)	Correlation between amino acid change and resistance to gentamicin (Spearman’s correlation coefficient *R*; *p*-value)	Correlation between amino acid change and resistance to tobramycin (Spearman’s correlation coefficient *R*; *p*-value)
*aph(3’’)-Ib*	-	60:5.3	< 0.01	0.77; < 0.01	0.54; < 0.01
*aph(6)- Id*	-	55:0	< 0.01	0.75; < 0.01	0.64; < 0.01
*armZ*	Cys40Arg	85:16	< 0.01	0.61; < 0.01	0.36; < 0.01
	Ser112Asn	85:5	< 0.01	0.68; < 0.01	0.44; < 0.01
	Asp119Glu	75:5	< 0.01	0.75; < 0.01	0.52; < 0.01
	Asp161Gly	20:95	< 0.01	-0.71; < 0.01	-0.48; < 0.01
	His182Gln	20:79	< 0.01	-0.49; < 0.01	-0.36; < 0.01
	Ile237Val	75:11	< 0.01	0.71; < 0.01	0.48; < 0.01
*parR*	Leu153Arg	80:16	< 0.01	0.53; < 0.01	*0.29; 0.07*
	Ser170Asn	85:11	< 0.01	0.64; < 0.01	0.40; 0.01
*mexX*	Ala30Thr	75:5	< 0.01	0.75; < 0.01	0.52; < 0.01
*mexY*	Ile536Val	70:0	< 0.01	0.72; < 0.01	0.60; < 0.01
	Gly589Ala	35:0	< 0.01	0.74; < 0.01	0.65; < 0.01
	Gln840Glu	45:0	< 0.01	0.62; < 0.01	0.64; < 0.01
	Asn1036Thr	35:0	< 0.01	0.68; < 0.01	0.58; < 0.01
*mexZ*	Gly89Ser	35:0	< 0.01	0.75; < 0.01	0.54; < 0.01

*Italicised* numbers indicate *p* > 0.05

In case of ArmZ, several amino acid changes showed significant differences (*p* < 0.01) in frequency between *exoU* and *exoS* strains (Table 3). Some of these amino acid changes exhibited moderate to strong positive correlations with resistance to gentamicin and tobramycin (Table 3), except Asp161Gly and His182Gln which were more common in *exoS* group and had negative correlations with resistance to gentamicin and tobramycin. SNPs that led to amino acid changes in ParR (Ser170Asn, Leu153Arg) were more frequent in *exoU* and correlated with resistance to gentamicin while in case of tobramycin, Ser170Asn showed moderate association and Leu153Arg showed no significant correlation. In efflux pump proteins, MexX Ala30Thr, MexY Ile536Val, Gly589Ala, Gln840Glu, Asn1036Thr, and MexZ Gly89Ser were more common in *exoU*. These mutations were positively correlated with gentamicin and tobramycin resistance (Table 3).

If strains contained mutations in *armZ*,* parR*,* mexX*,* mexY* and *mexZ* leading to amino acid changes they were more likely to be associated with extremely high resistance to aminoglycosides. For instance, PA31, PA32, PA33, PA35, PA37, PA219 and PA221 having mutations in all these genes showed MICs of 2560 µg/mL to ≥ 5120 µg/mL for gentamicin and 640 µg/mL to ≥ 5120 for tobramycin (Supplementary Table 8).

### Association of *exoU* and *exoS* and mutations in the DNA mismatch repair system genes

Mutations in genes involved in the DNA mismatch repair (MMR) system of 20 *exoU* and 19 *exoS* were examined for their prevalence and associations with mutations in AMR-associated genes (Table 4, Supplementary Table 9). Isolates harboring *exoU* displayed medians of one SNP in *mutL* (interquartile range (IQR) = 1 − 0), no SNPs in *mutS* (IQR = 1 − 0), and four SNPs in *uvrD* (IQR = 3.5-5). Conversely, *exoS*-containing isolates showed medians of zero SNP in *mutL* (IQR = 0), zero in *mutS* (IQR = 0), and two SNPs in *uvrD* (IQR = 2 − 0). Statistically significant differences were observed between *exoU* and *exoS* isolates in the number of SNPs in *mutL* (70% vs. 15.8%, *p* < 0.01) and *mutS***(**45% vs. 5.3%, *p* < 0.01**)**, but not for *uvrD* (90% vs. 63.2%, *p* = 0.06).

A crude analysis of correlations between the total number of SNPs in AMR associated genes with numbers in *mutL*, *mutS*, *uvrD* or total for all *mut* genes revealed significant correlations (Table 4). Number of SNPs in *mutL* (*R* = 0.47; *p* < 0.01), *mutS* (*R* = 0.43; *p* < 0.01), *uvrD* (*R* = 0.45; *p* < 0.01) and total number of SNPs in *mut* (*R* = 0.55; *p* < 0.01) genes correlated with total number of SNPs in AMR-associated genes.

**Table 4:** SNPs in the DNA mismatch repair (MMR) system genes of 20 *ExoU* and 19 *exos* keratitis isolates

Strains ID	TTSS group	mutL	mutS	uvrD	Total number of SNPs in mut genes^1^	Total number of SNPs in AMR-associated genes^2^
PA31	*exoU*	1	0	4	5	54
PA32	*exoU*	1	0	4	5	54
PA33	*exoU*	1	0	4	5	54
PA34	*exoU*	0	0	3	3	53
PA35	*exoU*	1	0	4	5	54
PA37	*exoU*	1	0	4	5	54
PA82	*exoU*	0	1	5	6	43
PA123	*exoU*	0	0	0	0	33
PA126	*exoU*	0	0	0	0	32
PA127	*exoU*	1	0	1	2	32
PA162	*exoU*	1	0	5	6	55
PA169	*exoU*	0	1	5	6	41
PA175	*exoU*	2	1	5	8	51
PA198	*exoU*	1	1	4	6	53
PA202	*exoU*	1	1	5	7	51
PA217	*exoU*	1	1	4	6	48
PA219	*exoU*	1	1	4	6	54
PA220	*exoU*	1	1	5	7	49
PA221	*exoU*	1	1	5	7	51
PA233	*exoU*	0	0	2	2	51
PA17	*exoS*	0	0	2	2	36
PA40	*exoS*	0	0	0	0	33
PA149	*exoS*	0	0	2	2	35
PA157	*exoS*	0	0	2	2	35
PA171	*exoS*	0	0	0	0	32
PA176	*exoS*	1	0	1	2	30
PA181	*exoS*	0	0	0	0	33
PA182	*exoS*	1	0	2	3	32
PA188	*exoS*	0	0	2	2	33
PA189	*exoS*	0	0	2	2	33
PA193	*exoS*	0	0	0	0	35
PA206	*exoS*	0	1	0	1	79
PA216	*exoS*	0	0	0	0	37
PA218	*exoS*	0	0	0	0	31
PA223	*exoS*	0	0	2	2	41
PA224	*exoS*	1	0	2	3	37
PA225	*exoS*	0	0	5	5	31
PA227	*exoS*	0	0	5	5	30
PA235	*exoS*	0	0	1	1	32

^1,^*mut* genes are *mutL*, *mutS* and *uvrD*; ^2,^ AMR-associated genes are *- gyrA*, *parC*, *parE*, *mexR*, *nalC*, *nalD*, *mexT*, *mexS*, *mexZ*, *mexA*, *mexB*, *oprM*, *mexC*, *mexD*, *oprJ*, *mexE*, *mexF*, *oprN*, *mexX*, *mexY*, *armZ*, *parR*, and *parS*

### Comparison of phenotypic antibiotic resistance between Australian and Indian keratitis isolates

Comparative analysis of antibiotic resistance between Australian (132) and (55) Indian isolates showed that Indian isolates were more resistant to all tested antibiotics than the Australian isolates (Supplementary Table 10). Resistance to ciprofloxacin (43.6% vs. 18.2%, *p* < 0.01), levofloxacin (27.3% vs. 12.9%, *p* = 0.03), and tobramycin (29.1% vs. 13.6%, *p* = 0.02) was significantly higher in Indian isolates, while gentamicin resistance was slightly but not significantly higher (32.7% vs. 23.5%; *p* = 0.20) (Supplementary Table 10). The antibiotic resistance of whole genome sequenced Australian (20) and Indian (19) isolates was compared, and the results showed that Indian isolates had significantly higher resistance to all tested antibiotics than the Australian isolates (*p* < 0.05) (Supplementary Table 11).

### Association between antibiotics resistance (fluroquinolone and aminoglycosides) and SNPs in efflux pumps, their regulatory genes, DNA mismatch repair system genes and possession of acquired resistance genes of *exoU* and *exoS* Indian keratitis isolates

A higher resistance rate among the whole genome sequenced Indian isolates compared to the Australian isolates against the four tested antibiotics appears to be largely driven by the 13 *exoU* Indian isolates (as compared to 6 *exoU* Australian strains), which exhibited a higher resistance to all antibiotics. Given the substantial impact of Indian isolates on overall resistance patterns, a detailed comparative analysis of SNPs variations and acquired resistance gene prevalence between 13 *exoU* and 6 *exoS* Indian strains were conducted to examine associations of significantly different mutations in *exoU* strains with antibiotics resistance.

The analysis of the Indian 13 *exoU* and 6 *exoS* isolates revealed similar patterns of SNPs possession to those observed in the broader 39-strains analysis (20 *exoU* and 19 *exoS*), with significant correlations between specific mutations and resistance to fluoroquinolones, gentamicin, and tobramycin (Supplementary Tables 12 and 13). Mutations in GyrA (Thr83Ile), ParC (Ser87Ile), NalC (Asp79Glu), MexB (Ser1041Glu, Val1042Ala) were exclusively present in *exoU* strains, with strong correlations (R ranging from 0.51 to 0.79; *p* ≤ 0.03) with increased resistance to ciprofloxacin and levofloxacin. Similarly, mutations in MexC (Glu251Gln, Ala262Glu, Ala277Thr) and MexD (Thr87Ser) correlated with levofloxacin resistance in *exoU* strains (Supplementary Table 12).

For aminoglycoside resistance, high frequencies of *aph(3’’)-Ib* and *aph(6)-Id* genes were present only in *exoU* strains and corresponded to significant associations with resistance to gentamicin and tobramycin (Supplementary Table 13). Additionally, mutations in ArmZ (Cys40Arg, Ser112Asn, Asp119Glu, Ile237Val), MexX (Ala30Thr) and MexY (Ile536Val, Gly589Ala, Gln840Glu, Asn1036Thr) showed moderate to strong positive correlations with aminoglycoside resistance (Supplementary Table 13).

Differences in SNPs prevalence in the DNA MMR system genes (*mutL* and *uvrD*) also showed significantly greater proportion of SNPs in 13 *exoU* than the 6 *exoS* Indian isolates (Supplementary Tables 14 and 15) indicating similar pattern of the 39 strains.

## Discussion

This study reports in vitro phenotypic antibiotic resistant pattern differences between *exoU* and *exoS P. aeruginosa* isolates from MK, and comparative analyses of the genetic make of *exoU* and *exoS* isolates to find associations with the phenotypic resistance. The overall, or within the Australian or Indian isolates, prevalence of *exoS* (71%) was higher than the *exoU* (29%). This result is consistent with a previous study where 64% keratitis isolates possessed the *exoS* and 33% carried the *exoU* [26]. These findings contrast with a previous study from the United Kingdom in which *exoU* strains (59%) were more prevalent than *exoS* strains (38%) [49]. However, they align with other reports indicating that *exoS* strains are generally more common than *exoU* strains in keratitis cases [10, 16, 17]. As ciprofloxacin (or other fluoroquinolones) or an aminoglycoside (e.g. gentamicin; sometimes fortified with a cephalosporin) are often used as the first line therapy for treatment of MK [20, 21], genetic changes in genes encoding for resistance to these antibiotics were examined.

Previous studies had shown that *exoS* and *exoU* strains possess different antibiotic resistance patterns [25, 26]. In the current study, *exoU* isolates had significantly higher resistance (MICs) to fluoroquinolones and aminoglycosides compared to *exoS* strains. These observations align with other studies [24, 25, 50, 51, 52], where *exoU* strains were generally more resistant to ciprofloxacin, gentamicin and tobramycin or resistance was more common compared to *exoS*. The current study found many significant associations with amino acid changes or possession of acquired resistance genes with possession of *exoU* and associations with resistance, many of which have not been previously reported. Whilst these associations (correlations) do not necessarily imply causality, the findings do provide the basis for hypothesis-driven studies to examine causality.

The presence of mutations in *gyrA* and *parC* play pivotal roles in conferring fluoroquinolone resistance in *P. aeruginosa* [53, 54]. Mutations in *gyrB* and *parE* are less common but can contribute to resistance by affecting the functions of gyrase and topoisomerase IV [54, 55, 56]. Of the 20 *exoU* and 19 *exoS* keratitis strains, no strain possessed any mutation in the *gyrB*, whereas the Asp533Glu change in ParE was moderately correlated with ciprofloxacin resistance in the current study. The substitution of threonine at position 83 with isoleucine in GyrA results in reduction of the binding affinity of fluoroquinolones to DNA gyrase leading to reduced susceptibility [53]. The combination of Thr83Ile and Ser87Ile in GyrA and ParC significantly enhances resistance by impairing both DNA gyrase and topoisomerase IV binding, effectively preventing inhibition of DNA replication and repair by fluoroquinolone [54, 57, 58]. The lack of such mutations in *exoS* strains might be the reason for those isolates showing relatively low levels of resistance to fluoroquinolone. However, *exoS* strains, and only *exoS* strains, possessed novel functional mutations in GyrA (Asp652Tyr, Ala659Tyr) and often showed high resistance to ciprofloxacin and levofloxacin for the *exoS* strains. This indicates that these novel mutations might play a role in fluroquinolone resistance and producing knock-out strains will help elucidate the role of these genes.

The associations have been found in some other studies. The combination of *exoU* with Thr83Ile in GyrA and Ser87Ile in ParC was more common in isolates from a Korean hospital (but that did not reach statistical significance, *p* = 0.09) [25], as well as from respiratory isolates from France (*p* = 0.05) [52]. In isolates from bronchial secretions, urine, wounds, blood (and elsewhere) at two Peruvian hospitals, although the possession of *exoU* was associated with high levels of fluoroquinolone resistance, the association of *exoU* with Thr83Ile in GyrA, Ser87Ile in ParC was not statistically significant [59]. A study from Japan found that *exoU* isolates significantly overexpressed *mexB* (a component of the the MexAB-OprM efflux pump), but had reduced expression of *mexY* (a member of the MexXY efflux system), although the mutations responsible for these outcomes were not investigated [60].

The SNP in *mexR*, which is a repressor of the MexAB-OprM efflux pump, leading to Val126Glu has been found more frequently in *exoU* strains (*p* = 0.01) [59]. This mutation in *mexR* associated with *exoU* has been reported previously [24, 54]. A previous study of keratitis isolates demonstrated that *exoU* isolates that had SNPs leading to Glu114Ala, Gly283Glu, and Met288Arg in MexR and these were associated with reduced expression of *mexR* [24]. These changes were not found in the current study, where a Val126Glu change was observed. Examining the effects of this change on expression of *mexR* is warranted.

Mutations in NalD, (leading to Arg38Trp, Ser32Asn or the amino acid frame shift), were found only in four *exoU* resistant strains. The gene *nalD* is another repressor of MexAB-OprM [61], with the Ser32Asn linked to its over-expression [62]. As the Arg38Trp mutation found in the current study is novel, it is not known whether this affects MexAB-OprM. Asp79Glu in NalC was present only in *exoU* group and correlated with fluoroquinolones resistance. The gene *nalC* indirectly works as a repressor for MexAB-OprM efflux pump and mutations in *nalC* genes can impair their functions and increase in bacterial resistance [63, 64] through overexpression of MexAB-OprM pump [65]. Studies should be conducted to determine whether the amino acid changes found in the current study are involved in the resistance.

The SNP in *mexZ* leading to Gly89Ser correlated with ciprofloxacin and levofloxacin resistance. Mutations in *mexZ* can lead to overexpression of the MexXY-OprM efflux pump further contributing to fluoroquinolone resistance by increasing drug efflux and reducing intracellular antibiotic concentrations [64]. Val73Ala in MexS was present only in *exoU* group and was associated with fluoroquinolones resistance. MexS downregulates MexEF-OprN through negative regulation, however, mutations in MexS can lead to overexpression of the MexEF-OprN efflux pump [66] resulting in increased bacterial resistance to fluoroquinolones [63, 66].

Isolates having the acquired resistance genes *crpP* and *qnrVC1* and mutations in the *gyrA*, *parC*, *nalC* and *mexZ* genes showed elevated level resistance to ciprofloxacin and levofloxacin. This suggests that cumulative mutations in multiple resistance-associated genes and the effects of the acquired resistant genes are responsible for the exceptionally high resistance to fluoroquinolones of some strains [28, 54, 67, 68]. Whilst several novel mutations in efflux pump genes *mexB*, *mexC*, *mexD*, *oprN*, *oprJ*, *mexX* and *mexY* were correlated with fluoroquinolones resistance, except, Ala262Glu and Ala277Thr in MexC, and Ser13Pro in OprN, all the other mutations in the efflux pumps genes did not show any presumed functional changes in the protein. Further study is needed to confirm the effects of these new mutations in expression of the efflux pumps and association with the fluoroquinolone’s resistance. Interestingly, amino acid changes in OprJ (Asp68Gly and Met69Val), the outer membrane channel of the MexCD-OprJ multidrug efflux complex was more common in *exoS* isolates and correlated with increased susceptibility to both fluoroquinolones. This could indicate a greater ability to transport these through this efflux pump system, as for the Met69Val change at least, this is not associated with overexpression of the gene [69].

Resistance to aminoglycosides in *P. aeruginosa* is conferred by both acquired genes and mutations in the regulatory genes of the efflux pump MexXY-OprM [70]. As with other studies [35, 71], *aph(3’)-IIb* was present in all isolates regardless of resistance profile, indicating that it does not mediate resistance to gentamicin and tobramycin. The genes *aph(6)-Id* and *aph(3’’)-Ib* had significantly higher frequency in the *exoU* strains and correlated strongly with resistance to gentamicin and tobramycin. A previous study has also shown these genes are related to gentamicin and tobramycin resistance [70].

Mutations in *mexZ*, a repressor of genes for MexXY-OprM, can reduce the sensitivity to aminoglycosides by increasing the expression of MexXY-OprM [72, 73]. In the current study, a novel functional mutation in MexZ (Gly89Ser) was carried by 35% *exoU* group and showed strong correlations with aminoglycosides resistance. Previous studies have reported 21–77% of aminoglycoside resistant strains carry other mutations in *mexZ* [35, 74]. Mutations in *armZ* (an anti-repressor of *mexZ*) lead to expression of the MexXY-OprM efflux pump [35, 38, 39]. The amino acid changes Cys40Arg, Ser112Asn, Asp119Glu and Ile237Val in the current study were associated with resistance to both aminoglycosides, whereas the Asp161Gly, His182Gln changes were associated with susceptibility to the aminoglycosides. A previous study has also reported that at least one these Cys40Arg, Ser112Asn, Asp119Glu and Ile237Val mutations was linked to resistance against gentamicin and tobramycin [35]. Another regulator of MexXY-OprM is ParRS, a two-component regulatory system [40]. The amino acid changes in ParR (Ser170Asn and Leu153Arg) differed between *exoU* and *exoS* groups and a previous study has identified these two mutations as the most common in ParR [75]. Ser170Asn had association with both gentamicin and tobramycin while Leu153Arg showed correlation only with gentamicin resistance. Similar results were also found in another study, where the presence of amino acid changes in ParR was correlated with gentamicin resistance but not with tobramycin resistance [35]. The novel mutations in MexX (Ala30Thr) and MexY (Ile536Val, Gly589Ala, Gln840Glu and Asn1036Thr) were also positively association with aminoglycoside resistance and future experiments are required to confirm these mutations' effect on MexXY-OprM expression or activity and resistance to the aminoglycosides.

Hypermutation plays an important role in the evolution of multidrug resistant bacteria [76]. Development of hypermutations in *P. aeruginosa* is associated with mutations in the DNA mismatch repair (MMR) system. The MMR system in *P. aeruginosa* relies on a protein trimer composed of MutS, MutL, and UvrD, which plays an important role in correcting DNA errors and maintaining genomic integrity. The *mutS* gene encodes a protein that binds to DNA errors; *mutL* encodes a protein to work with *mutS* to activate another protein encoded by *uvrD*; the latter gene encodes a DNA helicase and is involved in DNA replication [76]. *ExoU* isolates had a significantly higher frequency of mutations in the DNA mismatch repair (MMR) genes *mutL* and *mutS*, indicating that these isolates are more prone to hypermutation. Particularly, all SNPs in *mutS* were present in the *exoU* isolates except for one strain from the *exoS* group (PA206) and SNPs in all of these genes were highly correlated with the mutations in other antibiotic resistance genes, contributing to the resistance phenotype. Conversely, *exoS* carried fewer MMR mutations and lower levels of mutations in the resistance-associated genes, highlighting a less mutagenic profile and lower resistance potential than the *exoU* isolates. This difference in mutational profiles suggests that the defects in the MMR system are associated with the higher mutational burden and antibiotic resistance in the *exoU* isolates. These findings extend initial findings with 23 keratitis isolates, which found that in *exoU* isolates 75% contained mutations in *mutL*, 50% in *mutS* and 67% in *uvrD*, compared to 29% (*p* = 0.02), 14% (*p* = 0.05) and 92% (*p* = 0.05) [44]. Comparative analysis of 13 *exoU* and 6 *exoS* Indian isolates revealed a similar pattern to the broader 39-strain analysis (20 *exoU* and 19 *exoS*) in terms of differences in SNPs and the possession of acquired resistance genes associated with fluoroquinolone and aminoglycoside resistance.

## Conclusion

*ExoS* strains were more susceptible to ciprofloxacin, levofloxacin, gentamicin, and tobramycin than *exoU* strains in MK. Higher levels of resistance in *exoU* keratitis isolates were associated with the possession of greater number of resistance genes and different mutations in the AMR associated genes, and DNA mismatch repair (MMR) system genes. These data provide the foundation for hypothesis-driven studies to determine whether the novel mutations observed contribute to resistance against fluoroquinolones and aminoglycosides.

## Electronic supplementary material

Below is the link to the electronic supplementary material.

Supplementary Material 1

Supplementary Material 2

Supplementary Material 3

Supplementary Material 4

Supplementary Material 5

Supplementary Material 6

Supplementary Material 7

Supplementary Material 8

Supplementary Material 9

Supplementary Material 10

Supplementary Material 11

Supplementary Material 12

Supplementary Material 13

Supplementary Material 14

Supplementary Material 15

Supplementary Material 16

## Data Availability

Data is provided within the manuscript or supplementary information files.
