# Supplementary material for: Differences in antimicrobial resistance between exoU and exoS isolates of Pseudomonas aeruginosa
Source: Eur J Clin Microbiol Infect Dis. 2025 Apr 22;44(7):1629–41. doi: 10.1007/s10096-025-05132-6 (PMC12241228; doi:10.1007/s10096-025-05132-6)
Supplement: Supplementary file 2 — Supplementary Material 2 [file 10096_2025_5132_MOESM2_ESM.docx]

Supplementary Table 6: Functional SNPs in efflux pump genes leading to amino acid changes of 20 *exoU* and 19 *exoS* keratitis isolates

| Strain ID | TTSS group | SNPs in efflux pumps genes | | | | | | | | | | |
| --- | --- | --- | --- | --- | --- | --- | --- | --- | --- | --- | --- | --- |
|  |  | *mexA* | *mexB* | *oprM* | *mexC^1^* | *mexD* | *oprJ* | *mexE* | *mexF* | *oprN* | *mexX^2^* | *mexY^3^* |
| PA219 | *exoU* |  | Gly957Asp, Ser1041Glu, Val1042Ala |  | Glu251Gln, Ala262Glu, Ala277Thr, His310Arg, Ala378Thr, Pro383Ser, Ala384Val | Thr87Ser, Val434Ala, Ser845Ala |  |  |  | Ser13Pro | Ala30Thr | Ile536Val, Gly589Ala, Gln840Glu, Asn1036Thr |
| PA221 | *exoU* |  | Lys76Gln, Ser1041Glu, Val1042Ala |  | Ala31Val, Lys76Gln, His310Arg, Pro383Ser | Ala647Val, Ser845Ala |  | Ser8Phe | Asp606Glu | Ser13Pro | Ala30Thr | Ile536Val |
| PA198 | *exoU* |  | Gly957Asp, Ser1041Glu, Val1042Ala |  | Glu251Gln, Ala262Glu, Ala277Thr, His310Arg, Ala378Thr, Pro383Ser, Ala384Val | Thr87Ser, Ser845Ala |  |  |  | Ser13Pro | Ala30Thr | Ile536Val, Gly589Ala, Gln840Glu, Asn1036Thr |
| PA202 | *exoU* |  | Ser1041Glu, Val1042Ala |  | Ala31Val, Lys76Gln, His310Arg, Pro383Ser | Ala647Val, Ser845Ala |  | Ser8Phe | Asp606Glu | Ser13Pro | Ala30Thr | Ile536Val |
| PA33 | *exoU* |  | Gly957Asp, Ser1041Glu, Val1042Ala |  | Glu251Gln, Ala262Glu, Ala277Thr, His310Arg, Ala378Thr, Pro383Ser, Ala384Val | Thr87Ser, Ser845Ala |  |  |  | Ser13Pro | Ala30Thr | Ile536Val, Gly589Ala, Gln840Glu, Asn1036Thr |
| PA217 | *exoU* |  | Ser1041Glu, Val1042Ala |  | Lys76Gln, Ala262Glu, Pro383Ser | Glu257Gln, Ser845Ala, Lys1031Arg | Thr25Ile | Ser8Phe |  | Ser13Pro | Ala30Thr |  |
| PA32 | *exoU* |  | Gly957Asp, Ser1041Glu, Val1042Ala |  | Glu251Gln, Ala262Glu, Ala277Thr, His310Arg, Ala378Thr, Pro383Ser, Ala384Val | Thr87Ser, Ser845Ala |  |  |  | Ser13Pro | Ala30Thr | Ile536Val, Gly589Ala, Gln840Glu, Asn1036Thr |
| PA35 | *exoU* |  | Gly957Asp, Ser1041Glu, Val1042Ala |  | Glu251Gln, Ala262Glu, Ala277Thr, His310Arg, Ala378Thr, Pro383Ser, Ala384Val | Thr87Ser, Ser845Ala |  |  |  | Ser13Pro | Ala30Thr | Ile536Val, Gly589Ala, Gln840Glu, Asn1036Thr |
| PA37 | *exoU* |  | Gly957Asp, Ser1041Glu, Val1042Ala |  | Glu251Gln, Ala262Glu, Ala277Thr, His310Arg, Ala378Thr, Pro383Ser, Ala384Val | Thr87Ser, Ser845Ala |  |  |  | Ser13Pro | Ala30Thr | Ile536Val, Gly589Ala, Gln840Glu, Asn1036Thr |
| PA82 | *exoU* |  |  |  | Lys76Gln, Thr175Ala, His310Arg, Glu347Lys, Pro383Ser | Thr286Met, Ser845Ala |  |  | Asp230Ala | Ser13Pro | Ala30Thr |  |
| PA31 | *exoU* |  | Gly957Asp, Ser1041Glu, Val1042Ala |  | Glu251Gln, Ala262Glu, Ala277Thr, His310Arg, Ala378Thr, Pro383Ser, Ala384Val | Thr87Ser, Ser845Ala |  |  |  | Ser13Pro | Ala30Thr | Ile536Val, Gly589Ala, Gln840Glu, Asn1036Thr |
| PA233 | *exoU* |  |  |  | Ala262Glu, His310Arg, Val370Ala, Pro383Ser, Ala384Val | Glu257Gln, Asn775Lys, Ser845Ala, Lys1031Arg |  |  |  | Ser13Pro | Ala30Thr | Ile536Val |
| PA169 | *exoU* |  |  |  | Lys76Gln, Thr175Ala, His310Arg, Glu347Lys, Pro383Ser | Thr286Met, Ser845Ala |  |  |  | Ser13Pro |  | Ile536Val |
| PA220 | *exoU* |  | Ser1041Glu, Val1042Ala |  | Ala31Val, Lys76Gln, His310Arg, Pro383Ser | Ala647Val, Ser845Ala |  | Ser8Phe | Asp606Glu | Ser13Pro | Ala30Thr | Ile536Val |
| PA34 | *exoU* |  |  |  | His310Arg, Ala361Pro, Ala378Thr, Pro383Ser, Ala384Val | Ser845Ala, Ser915Ala, Ile982Val |  | Ser8Phe |  | Ser13Pro | Ala30Thr | Ile536Val, Gly589Ala, Gln840Glu, Asn1036Thr |
| PA123 | *exoU* |  |  |  | Ala328Val | Glu257Gln | Asp68Gly, Met69Val |  |  |  |  | Gln840Glu |
| PA127 | *exoU* |  |  |  | Ala328Val | Glu257Gln | Asp68Gly, Met69Val |  |  |  |  | Gln840Glu |
| PA126 | *exoU* | Lys289Arg |  |  | Val367Ala | Glu257Gln, Ser845Ala, Ala959Ser |  |  |  |  |  |  |
| PA162 | *exoU* |  |  |  | Ala378Thr,  Pro383Ser,  Ala384Val | Glu257Gln, Ser845Ala | Met69Val | Ser8Phe |  | Ser13Pro | Ala30Thr | Ile536Val, Gly589Ala, Gln840Glu, Asn1036Thr |
| PA175 | *exoU* | Lys76Gln |  |  | Lys76Gln, Ser313Gly, Pro383Ser | Ser845Ala, Lys1031Arg |  | Ser8Phe, Asp353Glu, Asp370Glu | Asp230Ala | Ser13Pro |  | Asn1036Thr |
| PA227 | *exoS* |  |  |  |  | Thr304Ala, Ser845Ala |  |  |  |  | Val331Leu |  |
| PA225 | *exoS* |  |  |  |  | Thr304Ala, Ser845Ala | Met69Val |  |  |  | Val331Leu |  |
| PA216 | *exoS* |  |  |  | His310Arg, Pro383Ser | Gln149His, Glu257Gln, Thr304Ala, Ser845Ala | Met69Val |  |  | Ser13Pro, Thr37Ala |  |  |
| PA223 | *exoS* |  |  |  |  | Glu257Gln, Ser845Ala | Asp68Gly, Met69Val |  |  |  |  | Asn1036Thr |
| PA224 | *exoS* |  |  |  |  |  |  |  |  |  |  | Gln840Glu |
| PA235 | *exoS* |  |  |  |  | Glu257Gln, Ser845Ala | Met69Val |  |  | Gly16Ser |  |  |
| PA218 | *exoS* |  |  |  | Ser313Gly | Ser845Ala |  |  |  | Ser13Pro |  |  |
| PA171 | *exoS* |  |  |  | Glu326Gly | Glu257Gln, Ser845Ala, Lys1031Arg |  |  |  |  |  |  |
| PA40 | *exoS* | Asp373Glu |  |  |  | Glu257Gln, Ile960Leu | Asp68Gly, Met69Val |  |  | Ser13Pro |  |  |
| PA17 | *exoS* |  |  |  | His310Arg, Ala378Thr, Pro383Ser, Ala384Val | Thr87Ser, Glu257Gln, Ser845Ala | Met69Val | Pro397Gln |  |  |  |  |
| PA188 | *exoS* |  |  |  |  | Glu257Gln, Ser845Ala | Asp68Gly, Met69Val |  |  | Ser13Pro | Val331Leu | Ala254Gly |
| PA206 | *exoS* |  |  |  | Ala31Thr, Lys76Gln, Asp298Ala, His310Arg, Pro383Ser, Ala384Val | Val364Leu, Ile610Val, Glu650Gln, Asn669Asp, Ser685Gly, Val687Leu, Ser845Ala, Ser915Ala, Ala1039Val | Phe6Leu, Ala30Val, Gly120Ser, Ala163Thr, Gly184Ser, Ser264Asn, Asp351Ala |  |  | Ser13Pro, Leu324Phe | Leu12Pro, Ala30Thr, Gly344Asp | Gln840Glu, Gly1035Asp, Asn1036Thr, Ile1040Thr |
| PA193 | *exoS* | Asp373Asn |  |  | Lys76Gln, His310Arg, | Glu257Gln, Ser845Ala |  |  |  | Ser13Pro |  |  |
| PA181 | *exoS* |  |  |  | His310Arg, Pro383Ser | Ser845Ala | Asp68Gly, Met69Val |  |  |  |  | Gln840Glu |
| PA182 | *exoS* |  |  |  |  | Ile960Leu | Met69Val |  | Ala598Glu |  |  |  |
| PA149 | *exoS* |  |  | Lys480Asn | Lys76Gln, Pro383Ser | Ala536Ser, Ser845Ala | Asp68Gly, Met69Val |  |  | Ala87Ser |  |  |
| PA176 | *exoS* |  |  |  |  | Glu257Gln, Ser281Leu, Ser845Ala | Met69Val |  |  |  |  |  |
| PA189 | *exoS* |  |  |  |  | Ser845Ala | Asp68Gly, Met69Val |  |  | Ser13Pro | Val331Leu | Ala254Gly |
| PA157 | *exoS* | Asp373Glu |  | Val72Leu |  | Ser845Ala | Asp68Gly, Met69Val |  |  |  |  | Val240Met, Gly508Ser, Phe509Ser |

All mutations are new, red indicates functional SNPs, ^1^, all strains had Ser330Ala in *mexC,* so not shown in the table; ^2^, all strains had Thr15Ala, Asp25Glu, Lys26Glu, Pro28Ala, Glu29Asp, Glu31Gly, Ala33Thr, Asp35Glu, Lys46Ar, Val133Ile, Ser153Ala, Arg161Lys, Gln236Lys, His338Arg, Gly384Asp, Val385Ala in *mexX*, so not shown in the table; ^3^, all strains had Thr543Ala in *mexY*, so not shown in the table.
