# Supplementary material for: Differences in antimicrobial resistance between exoU and exoS isolates of Pseudomonas aeruginosa
Source: Eur J Clin Microbiol Infect Dis. 2025 Apr 22;44(7):1629–41. doi: 10.1007/s10096-025-05132-6 (PMC12241228; doi:10.1007/s10096-025-05132-6)
Supplement: Supplementary file 3 — Supplementary Material 3 [file 10096_2025_5132_MOESM3_ESM.docx]

Supplementary Table 1. PCR primers and conditions for the amplification of *exoU* and *exoS*

| Gene | 5' to 3' primer sequence | PCR cycling conditions | Product size |
| --- | --- | --- | --- |
| *exoS* | F-GCGAGGTCAGCAGAGTATCG  R-TTCGGCGTCACTGTGGAT | Initial denaturation: 94°C for 2 min  94°C for 30 sec  36 cycles  58°C for 30 sec  68°C for 1 min  Final extension: 68°C for 7 min | 118 bp |
| *exoU* | F-ATGCATATCCAATCGTTG  R-TCATGTGAACTCCTTATT | Initial denaturation: 94°C for 5 min  94°C for 30 sec  36 cycles  57°C for 30 sec  72°C for 1 min  Final extension: 72°C for 10 min | 2000 bp |

Supplementary Table 2. Composition of the PCR reaction mixture

| 2X Green master mixture | 12.5 ul |
| --- | --- |
| Forward primer | 0.5 ul |
| Reverse primer | 0.5 ul |
| Template DNA | 1.5 ul |
| Water | upto 25 ul |
