# Supplementary material for: Differences in antimicrobial resistance between exoU and exoS isolates of Pseudomonas aeruginosa
Source: Eur J Clin Microbiol Infect Dis. 2025 Apr 22;44(7):1629–41. doi: 10.1007/s10096-025-05132-6 (PMC12241228; doi:10.1007/s10096-025-05132-6)
Supplement: Supplementary file 8 — Supplementary Material 8 [file 10096_2025_5132_MOESM8_ESM.docx]

Supplementary Table 9: Mutations in the DNA mismatch repair (MMR) system of 20 *exoU* and 19 *exoS* keratitis isolates

| Strains ID | **TTSS** | *mutL* | *mutS* | *uvrD* |
| --- | --- | --- | --- | --- |
| PA31 | *exoU* | Ala4Thr, Ala391Val |  | Val556Ile, Ala641Ser, Ser662Asn, Asn666Ser |
| PA32 | *exoU* | Ala4Thr, Ala391Val |  | Val556Ile, Ala641Ser, Ser662Asn, Asn666Ser |
| PA33 | *exoU* | Ala4Thr, Ala391Val |  | Val556Ile, Ala641Ser, Ser662Asn, Asn666Ser |
| PA34 | *exoU* |  |  | Ala641Ser, Ser662Asn, Asn666Ser |
| PA35 | *exoU* | Ala4Thr, Ala391Val |  | Val556Ile, Ala641Ser, Ser662Asn, Asn666Ser |
| PA37 | *exoU* | Ala4Thr, Ala391Val |  | Val556Ile, Ala641Ser, Ser662Asn, Asn666Ser |
| PA82 | *exoU* | Ala4Thr | Ala781Thr | Lys344Arg, Val556Ile, Ala641Ser, Ser662Asn, Asn666Ser |
| PA 123 | *exoU* |  |  |  |
| PA 126 | *exoU* |  |  |  |
| PA 127 | *exoU* | Pro345Leu |  | Ile237Val |
| PA 162 | *exoU* | Asn595Ser |  | Lys344Arg, Val556Ile, Ala641Ser, Ser662Asn, Asn666Ser |
| PA 169 | *exoU* | Ala4Thr | Ala781Thr | Lys344Arg, Val556Ile, Ala641Ser, Ser662Asn, Asn666Ser |
| PA175 | *exoU* | Arg370His, Ser515Asn | Ala781Thr | Lys344Arg, Val556Ile, Ala641Ser, Ser662Asn, Asn666Ser |
| PA198 | *exoU* | Ala4Thr, Ala391Val | Ala781Thr | Val556Ile, Ala641Ser, Ser662Asn, Asn666Ser |
| PA 202 | *exoU* | Ala391Val | Ala781Thr | Lys344Arg, Val556Ile, Ala641Ser, Ser662Asn, Asn666Ser |
| PA 217 | *exoU* | Ala4Thr, Ala391Val | Ala781Thr | Lys344Arg, Ala417Thr, Ser662Asn, Asn666Ser |
| PA 219 | *exoU* | Ala4Thr, Ala391Val | Ala781Thr | Val556Ile, Ala641Ser, Ser662Asn, Asn666Ser |
| PA 220 | *exoU* | Ala391Val | Ala781Thr | Lys344Arg, Val556Ile, Ala641Ser, Ser662Asn, Asn666Ser |
| PA 221 | *exoU* | Ala391Val | Ala781Thr | Lys344Arg, Val556Ile, Ala641Ser, Ser662Asn, Asn666Ser |
| PA 233 | *exoU* |  |  | Val556Ile, Ser662Asn |
| PA17 | *exoS* |  |  | Ser662Asn, Asn666Ser |
| PA40 | *exoS* |  |  |  |
| PA149 | *exoS* |  |  | Ser662Asn, Asn666Ser |
| PA157 | *exoS* |  |  | Ser662Asn, Asn666Ser |
| PA171 | *exoS* |  |  |  |
| PA 176 | *exoS* | Glu428Asp |  | Gly657Ala |
| PA 181 | *exoS* |  |  |  |
| PA 182 | *exoS* | Ala 391Val |  | Ser662Asn, Asn666Ser |
| PA 188 | *exoS* |  |  | Ser662Asn, Asn666Ser |
| PA 189 | *exoS* |  |  | Ser662Asn, Asn666Ser |
| PA 193 | *exoS* |  |  |  |
| PA 206 | *exoS* |  | Ser281Gly |  |
| PA 216 | *exoS* |  |  |  |
| PA 218 | *exoS* |  |  |  |
| PA 223 | *exoS* |  |  | Ser662Asn, Asn666Ser |
| PA 224 | *exoS* | Ala426Thr |  | Ser662Asn, Asn666Ser |
| PA 225 | *exoS* |  |  | Lys344Arg, Val556Ile, Ala641Ser, Ser662Asn, Asn666Ser |
| PA 227 | *exoS* |  |  | Lys344Arg, Val556Ile, Ala641Ser, Ser662Asn, Asn666Ser |
| PA 235 | *exoS* |  |  | Asn666Ser |
