# Supplementary material for: Differences in antimicrobial resistance between exoU and exoS isolates of Pseudomonas aeruginosa
Source: Eur J Clin Microbiol Infect Dis. 2025 Apr 22;44(7):1629–41. doi: 10.1007/s10096-025-05132-6 (PMC12241228; doi:10.1007/s10096-025-05132-6)
Supplement: Supplementary file 10 — Supplementary Material 10 [file 10096_2025_5132_MOESM10_ESM.docx]

Supplementary Table 3: Antimicrobial susceptibility of 55 *exoU* and 132 *exoS* keratitis isolates against ciprofloxacin, levofloxacin, gentamicin and tobramycin

| Country | Year of isolation | T3SS Type | Strain ID | Ciprofloxacin | Levofloxacin | Gentamicin | Tobramycin |
| --- | --- | --- | --- | --- | --- | --- | --- |
|  |  |  |  | Breakpoint  ≤1,2, ≥ 4 µg/ml | Breakpoint  ≤2, 4, ≥ 8 µg/ml | Breakpoint≤4, 8, ≥ 16 µg/ml | |
| Australia | 1994 | *exoU* | PA2 | 0.5 | 0.5 | 1 | 1 |
| Australia | 1994 | *exoU* | PA4 | 0.5 | 0.5 | 1 | 1 |
| Australia | 1994 | *exoU* | PA9 | 0.5 | 1 | 2 | 1 |
| Australia | 1994 | *exoU* | PA10 | 0.5 | 1 | 2 | 1 |
| Australia | 1995 | *exoU* | PA22 | 0.25 | 1 | 4 | 1 |
| Australia | 1995 | *exoU* | PA23 | 0.5 | 1 | 1 | 1 |
| Australia | 1998 | *exoU* | PA26 | 0.5 | 1 | 1 | 1 |
| Australia | 1998 | *exoU* | PA27 | 0.5 | 1 | 4 | 2 |
| Australia | 1998 | *exoU* | PA30 | 0.25 | 0.5 | 2 | 1 |
| India | 1998 | *exoU* | PA31 | **32** | **32** | **≥5120** | **640** |
| India | 1998 | *exoU* | PA32 | **64** | **32** | **2560** | **640** |
| India | 1998 | *exoU* | PA33 | **128** | **32** | **2560** | **≥5120** |
| India | 1998 | *exoU* | PA34 | 2 | 2 | **2560** | **640** |
| India | 1998 | *exoU* | PA35 | **64** | **32** | **2560** | **1280** |
| India | 1998 | *exoU* | PA37 | **64** | **32** | **2560** | **1280** |
| India | 1998 | *exoU* | PA38 | 0.5 | 1 | 2 | 1 |
| Australia | 2002 | *exoU* | PA46 | 0.5 | 0.5 | 1 | 1 |
| Australia | 2002 | *exoU* | PA48 | 0.5 | 0.5 | 1 | 1 |
| Australia | 2002 | *exoU* | PA49 | 0.5 | 0.5 | 1 | 1 |
| Australia | 2004 | *exoU* | PA70 | 0.5 | 1 | 1 | 1 |
| Australia | 2004 | *exoU* | PA71 | 0.5 | 1 | 2 | 2 |
| India | 2004 | *exoU* | PA76 | **512** | **4** | **16** | **16** |
| India | 2004 | *exoU* | PA79 | 0.25 | 1 | 2 | 2 |
| India | 2004 | *exoU* | PA80 | 0.25 | 1 | 2 | 2 |
| India | 2004 | *exoU* | PA81 | 0.5 | 0.5 | **8** | 2 |
| India | 2004 | *exoU* | PA82 | **64** | **4** | **8** | 0.25 |
| Australia | 2005 | *exoU* | PA117 | 0.25 | 0.5 | **8** | 1 |
| Australia | 2005 | *exoU* | PA123 | 1 | 1 | 0.25 | 4 |
| Australia | 2005 | *exoU* | PA126 | 0.5 | 0.5 | 0.5 | 0.25 |
| Australia | 2005 | *exoU* | PA127 | 1 | 0.25 | 2 | 32 |
| Australia | 2006 | *exoU* | PA128 | 0.5 | 1 | 4 | 2 |
| Australia | 2006 | *exoU* | PA137 | 0.25 | 2 | **8** | 1 |
| Australia | 2006 | *exoU* | PA139 | 0.25 | 1 | **8** | 1 |
| Australia | 2006 | *exoU* | PA148 | 1 | 1 | **16** | **8** |
| Australia | 2006 | *exoU* | PA150 | **2** | **8** | **16** | **8** |
| Australia | 2006 | *exoU* | PA153 | 0.5 | 2 | **8** | 2 |
| Australia | 2006 | *exoU* | PA154 | 0.25 | 1 | **8** | 2 |
| Australia | 2006 | *exoU* | PA159 | 0.5 | 2 | **512** | **512** |
| Australia | 2006 | *exoU* | PA162 | 0.5 | 0.5 | 0.25 | 0.25 |
| Australia | 2006 | *exoU* | PA169 | **2** | 0.25 | 0.25 | 0.25 |
| Australia | 2006 | *exoU* | PA175 | 0.25 | 0.25 | 0.25 | 0.25 |
| India | 2017 | *exoU* | PA198 | **1280** | **320** | **2560** | **16** |
| India | 2017 | *exoU* | PA200 | **32** | **16** | 0.25 | 0.25 |
| India | 2017 | *exoU* | PA202 | **640** | **320** | **8** | **320** |
| India | 2017 | *exoU* | PA204 | 0.5 | 0.5 | 0.5 | 0.25 |
| India | 2017 | *exoU* | PA210 | **≥5120** | **≥5120** | 4 | 4 |
| India | 2018 | *exoU* | PA217 | **64** | **32** | 1 | 1 |
| India | 2018 | *exoU* | PA219 | **≥5120** | **640** | **≥5120** | **1280** |
| India | 2018 | *exoU* | PA220 | **2** | 0.25 | 0.5 | 0.5 |
| India | 2018 | *exoU* | PA221 | **2560** | **2560** | **2560** | **2560** |
| Australia | 2018 | *exoU* | PA228 | 0.5 | 0.25 | 0.25 | 0.25 |
| Australia | 2018 | *exoU* | PA229 | 0.25 | 2 | 0.5 | 0.25 |
| Australia | 2019 | *exoU* | PA233 | **8** | 1 | 1 | 0.5 |
| Australia | 2019 | *exoU* | PA234 | 0.5 | 1 | 4 | 2 |
| Australia | 2022 | *exoU* | AkPA1 | **256** | **256** | **256** | **256** |
| Australia | 1993 | *exoS* | PA1 | 0.5 | 0.5 | 1 | 1 |
| Australia | 1994 | *exoS* | PA3 | 0.5 | 0.5 | 1 | 1 |
| Australia | 1994 | *exoS* | PA5 | 0.5 | 0.5 | 1 | 1 |
| Australia | 1994 | *exoS* | PA7 | 0.5 | 1 | 4 | 1 |
| Australia | 1994 | *exoS* | PA8 | 0.5 | 1 | 2 | 1 |
| Australia | 1994 | *exoS* | PA11 | 0.5 | 1 | 2 | 1 |
| Australia | 1994 | *exoS* | PA12 | 0.5 | 1 | 2 | 1 |
| Australia | 1994 | *exoS* | PA13 | 0.5 | 1 | 2 | 1 |
| Australia | 1994 | *exoS* | PA14 | 0.5 | 1 | 2 | 1 |
| Australia | 1994 | *exoS* | PA15 | 0.5 | 1 | 2 | 1 |
| Australia | 1994 | *exoS* | PA16 | 0.5 | 1 | 2 | 1 |
| Australia | 1994 | *exoS* | PA17 | **2** | 1 | 0.25 | 0.25 |
| Australia | 1994 | *exoS* | PA18 | 0.25 | 0.5 | 4 | 2 |
| Australia | 1995 | *exoS* | PA21 | 0.5 | 1 | 4 | 1 |
| Australia | 1997 | *exoS* | PA24 | 0.5 | 1 | 1 | 1 |
| India | 1997 | *exoS* | PA25 | 0.5 | 1 | 1 | 1 |
| Australia | 1998 | *exoS* | PA28 | 0.5 | 2 | 4 | **8** |
| Australia | 1998 | *exoS* | PA29 | 0.25 | 1 | 4 | 2 |
| India | 1998 | *exoS* | PA36 | 0.5 | 1 | 2 | 1 |
| India | 1998 | *exoS* | PA39 | 0.5 | 1 | 2 | 1 |
| Australia | 1999 | *exoS* | PA40 | **4** | 2 | 0.25 | 0.25 |
| Australia | 1999 | *exoS* | PA41 | 0.5 | 1 | 2 | 1 |
| Australia | 2001 | *exoS* | PA43 | 0.25 | 1 | **8** | 1 |
| Australia | 2001 | *exoS* | PA44 | 1 | 1 | 4 | 2 |
| Australia | 2002 | *exoS* | PA45 | 0.5 | 0.5 | 1 | 1 |
| Australia | 2002 | *exoS* | PA47 | 0.5 | 0.5 | 1 | 1 |
| Australia | 2003 | *exoS* | PA50 | **2** | **4** | **32** | 4 |
| Australia | 2003 | *exoS* | PA51 | 1 | 2 | **512** | **128** |
| Australia | 2003 | *exoS* | PA52 | 0.5 | 1 | **128** | **16** |
| Australia | 2003 | *exoS* | PA53 | **2** | **8** | 2 | 1 |
| Australia | 2003 | *exoS* | PA54 | 0.5 | 0.5 | **512** | **128** |
| Australia | 2003 | *exoS* | PA56 | **2** | **4** | **512** | **256** |
| Australia | 2003 | *exoS* | PA58 | 1 | 2 | **256** | **64** |
| Australia | 2003 | *exoS* | PA60 | 0.25 | 0.25 | **64** | **16** |
| Australia | 2003 | *exoS* | PA61 | **32** | **64** | **256** | **64** |
| Australia | 2003 | *exoS* | PA62 | 4 | **8** | **256** | **64** |
| Australia | 2003 | *exoS* | PA63 | 1 | 2 | 4 | 2 |
| Australia | 2003 | *exoS* | PA65 | **8** | **16** | **512** | **256** |
| Australia | 2003 | *exoS* | PA67 | 0.25 | 1 | **64** | **16** |
| Australia | 2003 | *exoS* | PA69 | **8** | **16** | **1024** | **256** |
| India | 2004 | *exoS* | PA72 | 0.25 | 1 | **8** | 4 |
| India | 2004 | *exoS* | PA73 | 0.5 | **4** | **16** | **16** |
| India | 2004 | *exoS* | PA74 | 0.25 | 2 | **8** | 4 |
| India | 2004 | *exoS* | PA75 | **512** | 1 | 4 | 2 |
| India | 2004 | *exoS* | PA77 | 0.25 | 1 | **8** | 4 |
| India | 2004 | *exoS* | PA78 | **8** | 2 | 2 | 2 |
| Australia | 2004 | *exoS* | PA108 | **4** | **8** | **8** | 2 |
| Australia | 2004 | *exoS* | PA110 | 0.5 | 1 | **512** | **128** |
| Australia | 2004 | *exoS* | PA111 | 0.25 | 1 | 4 | 2 |
| Australia | 2004 | *exoS* | PA112 | 0.25 | 1 | 4 | 2 |
| Australia | 2004 | *exoS* | PA113 | 0.25 | 0.25 | 2 | 1 |
| Australia | 2004 | *exoS* | PA114 | 0.25 | 0.5 | **8** | 2 |
| Australia | 2005 | *exoS* | PA115 | 0.25 | 0.5 | 1 | 0.25 |
| Australia | 2005 | *exoS* | PA116 | 0.25 | 0.5 | 0.5 | 0.25 |
| Australia | 2005 | *exoS* | PA119 | 0.5 | 1 | 2 | 4 |
| Australia | 2005 | *exoS* | PA120 | 0.25 | 1 | 4 | 1 |
| Australia | 2006 | *exoS* | PA121 | 0.25 | 1 | 0.5 | 2 |
| Australia | 2006 | *exoS* | PA122 | 1 | 1 | 4 | 2 |
| Australia | 2006 | *exoS* | PA124 | 1 | 0.5 | 0.5 | 0.25 |
| Australia | 2006 | *exoS* | PA125 | **2** | **4** | **8** | 2 |
| Australia | 2006 | *exoS* | PA129 | 0.25 | 0.25 | 0.25 | 0.25 |
| Australia | 2006 | *exoS* | PA134 | 0.5 | 2 | 4 | 2 |
| Australia | 2006 | *exoS* | PA135 | 0.5 | 1 | **8** | 4 |
| Australia | 2006 | *exoS* | PA136 | 0.5 | 1 | 4 | 2 |
| Australia | 2006 | *exoS* | PA138 | 1 | **4** | 4 | 1 |
| Australia | 2006 | *exoS* | PA140 | 0.25 | 1 | **8** | 2 |
| Australia | 2006 | *exoS* | PA141 | 0.25 | 0.5 | **8** | 2 |
| Australia | 2006 | *exoS* | PA142 | 0.25 | 0.5 | **8** | 4 |
| Australia | 2006 | *exoS* | PA144 | 0.25 | 0.5 | 2 | 2 |
| Australia | 2006 | *exoS* | PA145 | 0.5 | 2 | 4 | 1 |
| Australia | 2006 | *exoS* | PA147 | 1 | **4** | **8** | 2 |
| Australia | 2006 | *exoS* | PA149 | 0.5 | 0.5 | 0.25 | 0.25 |
| Australia | 2006 | *exoS* | PA151 | **2** | **8** | **32** | **16** |
| Australia | 2006 | *exoS* | PA152 | 0.5 | 1 | 4 | 1 |
| Australia | 2006 | *exoS* | PA155 | 0.25 | 0.25 | 0.25 | 4 |
| Australia | 2006 | *exoS* | PA156 | 0.5 | 1 | 4 | 2 |
| Australia | 2006 | *exoS* | PA157 | 0.25 | 0.5 | 0.25 | 0.25 |
| Australia | 2006 | *exoS* | PA160 | 1 | 0.5 | 4 | 2 |
| Australia | 2006 | *exoS* | PA163 | 0.5 | 1 | 4 | **16** |
| Australia | 2006 | *exoS* | PA164 | 0.5 | 2 | 4 | 2 |
| Australia | 2006 | *exoS* | PA165 | 1 | 0.25 | 0.25 | 0.25 |
| Australia | 2006 | *exoS* | PA166 | 1 | 2 | **8** | 4 |
| Australia | 2006 | *exoS* | PA167 | 1 | 2 | 4 | 4 |
| Australia | 2006 | *exoS* | PA170 | 0.5 | 0.5 | 0.5 | 2 |
| Australia | 2006 | *exoS* | PA171 | **4** | 2 | 0.25 | 0.25 |
| Australia | 2006 | *exoS* | PA172 | 0.5 | 0.5 | **8** | 2 |
| Australia | 2006 | *exoS* | PA173 | 1 | **4** | 4 | 2 |
| Australia | 2006 | *exoS* | PA174 | 0.25 | 0.25 | 0.25 | 0.25 |
| Australia | 2006 | *exoS* | PA176 | 0.5 | 0.25 | 0.25 | 0.25 |
| Australia | 2006 | *exoS* | PA177 | 1 | 1 | 2 | 2 |
| Australia | 2006 | *exoS* | PA178 | 0.5 | 1 | **512** | 1 |
| Australia | 2006 | *exoS* | PA179 | **2** | 0.25 | 0.25 | 0.5 |
| Australia | 2006 | *exoS* | PA180 | 0.5 | 0.5 | 4 | 1 |
| Australia | 2006 | *exoS* | PA181 | 1 | 0.25 | 0.25 | 0.25 |
| Australia | 2006 | *exoS* | PA182 | 1 | 0.25 | 0.25 | 0.25 |
| Australia | 2006 | *exoS* | PA183 | 0.5 | 0.5 | 4 | 1 |
| Australia | 2006 | *exoS* | PA184 | 0.5 | 0.5 | 2 | 1 |
| India | 2017 | *exoS* | PA188 | **2** | 1 | 0.5 | **32** |
| India | 2017 | *exoS* | PA189 | 0.25 | 1 | 0.25 | **16** |
| India | 2017 | *exoS* | PA190 | 0.25 | 0.5 | 0.25 | **8** |
| India | 2017 | *exoS* | PA191 | 1 | 1 | 0.25 | 2 |
| India | 2017 | *exoS* | PA192 | **2** | 0.25 | 0.5 | 0.25 |
| India | 2017 | *exoS* | PA193 | 1 | 0.25 | 0.25 | 0.25 |
| India | 2017 | *exoS* | PA194 | 0.5 | 0.5 | 1 | 0.25 |
| India | 2017 | *exoS* | PA195 | 0.5 | 0.5 | 0.5 | 0.25 |
| India | 2017 | *exoS* | PA196 | 0.5 | 0.25 | 0.5 | 0.25 |
| India | 2017 | *exoS* | PA197 | 1 | 0.25 | 0.5 | 0.25 |
| India | 2017 | *exoS* | PA199 | 0.25 | 0.25 | 0.25 | 0.25 |
| India | 2017 | *exoS* | PA201 | 1 | 0.25 | 0.25 | 0.5 |
| India | 2017 | *exoS* | PA203 | 0.25 | 1 | 0.25 | 0.5 |
| India | 2017 | *exoS* | PA205 | **2** | 0.5 | 0.25 | 0.25 |
| India | 2017 | *exoS* | PA206 | 1 | 0.5 | 1 | 0.25 |
| India | 2017 | *exoS* | PA207 | 0.25 | 0.5 | 1 | 0.25 |
| India | 2017 | *exoS* | PA208 | 0.5 | 0.25 | 0.5 | 0.25 |
| India | 2017 | *exoS* | PA209 | 1 | 1 | 1 | 0.5 |
| India | 2017 | *exoS* | PA211 | 0.5 | 0.25 | 0.25 | 0.25 |
| India | 2017 | *exoS* | PA212 | **8** | 1 | 0.5 | 0.25 |
| India | 2017 | *exoS* | PA213 | **2** | 1 | 1 | 0.25 |
| India | 2017 | *exoS* | PA215 | 0.5 | 1 | 1 | 0.5 |
| India | 2018 | *exoS* | PA216 | **64** | **4** | 1 | 0.5 |
| India | 2018 | *exoS* | PA218 | **8** | 1 | 0.5 | 0.5 |
| India | 2018 | *exoS* | PA222 | 0.25 | 2 | 2 | 2 |
| Australia | 2018 | *exoS* | PA223 | **64** | 1 | 0.5 | 0.5 |
| Australia | 2018 | *exoS* | PA224 | **16** | 1 | 0.25 | 0.25 |
| Australia | 2018 | *exoS* | PA225 | **64** | **16** | 0.5 | 0.25 |
| Australia | 2018 | *exoS* | PA226 | 1 | 1 | 1 | 1 |
| Australia | 2018 | *exoS* | PA227 | **64** | **64** | 0.5 | 0.25 |
| Australia | 2019 | *exoS* | PA230 | **4** | 2 | 2 | 0.5 |
| Australia | 2019 | *exoS* | PA231 | 1 | 2 | 4 | 2 |
| Australia | 2019 | *exoS* | PA232 | 0.5 | 1 | 4 | 2 |
| Australia | 2019 | *exoS* | PA235 | **16** | 0.5 | 2 | 0.5 |
| Australia | 2019 | *exoS* | PA320 | 0.5 | 2 | **8** | 2 |

Bold font = Resistant (including intermediate)
