# Supplementary material for: Differences in antimicrobial resistance between exoU and exoS isolates of Pseudomonas aeruginosa
Source: Eur J Clin Microbiol Infect Dis. 2025 Apr 22;44(7):1629–41. doi: 10.1007/s10096-025-05132-6 (PMC12241228; doi:10.1007/s10096-025-05132-6)
Supplement: Supplementary file 11 — Supplementary Material 11 [file 10096_2025_5132_MOESM11_ESM.docx]

Supplementary Table 8. Possession of acquired resistance genes and functional SNPs in genes leading to amino acid changes associated with aminoglycoside resistance of 20 *exoU* and 19 *exoS* keratitis isolates*

| Strains | TTSS group | MICs of Antibiotics (µg/ml);  Breakpoint≤4, 8, ≥ 16 | | Aminoglycosides acquired resistance genes | | | | | | | | | | | | Efflux pumps regulatory gene | | |
| --- | --- | --- | --- | --- | --- | --- | --- | --- | --- | --- | --- | --- | --- | --- | --- | --- | --- | --- |
|  |  | Gentamicin | Tobramycin | *aph(3')-Ib* | *aph(3')-Iib* | *aph(3')-VI* | *aph(3'')-Ib* | *aph(6)- Id* | *aac(3)-Iid* | *aac(6')-Ib3* | *aadA1* | *aadA10* | *aadA24* | *rmtD2* | *rmtB* | *armZ* | *parR* | *parS* |
|  |  |  |  |  |  |  |  |  |  |  |  |  |  |  |  |  |  |  |
|  |  |  |  |  |  |  |  |  |  |  |  |  |  |  |  |  |  |  |
| PA31 | *exoU* | **≥5120** | **640** |  |  |  |  |  |  |  |  |  |  |  |  | Cys40Arg, Ser112Asn, Asp119Glu, Ile237Val | Met59Ile, Leu153Arg, Ser170Asn |  |
| PA32 | *exoU* | **2560** | **640** |  |  |  |  |  |  |  |  |  |  |  |  | Cys40Arg, Ser112Asn, Asp119Glu, Ile237Val | Met59Ile, Leu153Arg, Ser170Asn |  |
| PA33 | *exoU* | **2560** | **≥5120** |  |  |  |  |  |  |  |  |  |  |  |  | Cys40Arg, Ser112Asn, Asp119Glu, Ile237Val | Met59Ile, Leu153Arg, Ser170Asn |  |
| PA34 | *exoU* | **2560** | **640** |  |  |  |  |  |  |  |  |  |  |  |  | Cys40Arg, Asp104Glu, Ser112Asn, Asp119Glu, His182Gln, Asp207Asn, Ile237Val | Ser170Asn | Arg243His, Gly388Asp |
| PA35 | *exoU* | **2560** | **1280** |  |  |  |  |  |  |  |  |  |  |  |  | Cys40Arg, Ser112Asn, Asp119Glu, Ile237Val | Met59Ile, Leu153Arg, Ser170Asn |  |
| PA37 | *exoU* | **2560** | **1280** |  |  |  |  |  |  |  |  |  |  |  |  | Cys40Arg, Ser112Asn, Asp119Glu, Ile237Val | Met59Ile, Leu153Arg, Ser170Asn |  |
| PA82 | *exoU* | **8** | 0.25 |  |  |  |  |  |  |  |  |  |  |  |  | Cys40Arg, Ser112Asn, Asp119Glu | Leu153Arg, Ser170Asn |  |
| PA123 | *exoU* | 0.25 | 4 |  |  |  |  |  |  |  |  |  |  |  |  | Asp161Gly, His182Gln | Ile93Thr |  |
| PA126 | *exoU* | 0.5 | 0.25 |  |  |  |  |  |  |  |  |  |  |  |  | Asp161Gly, His182Gln |  |  |
| PA127 | *exoU* | 2 | 32 |  |  |  |  |  |  |  |  |  |  |  |  | Asp161Gly, His182Gln | Ile93Thr |  |
| PA162 | *exoU* | 0.25 | 0.25 |  |  |  |  |  |  |  |  |  |  |  |  | Cys40Arg, Ser112Asn, Asn238Ser | Leu153Arg, Ser170Asn, Gly232Asp |  |
| PA169 | *exoU* | 0.25 | 0.25 |  |  |  |  |  |  |  |  |  |  |  |  | Cys40Arg, Ser112Asn, Asp119Glu, Ile237Val | Leu153Arg, Ser170Asn | Ser277Asn |
| PA175 | *exoU* | 0.25 | 0.25 |  |  |  |  |  |  |  |  |  |  |  |  | Cys40Arg, Ser112Asn | Leu153Arg, Ser170Asn |  |
| PA198 | *exoU* | **2560** | **16** |  |  |  |  |  |  |  |  |  |  |  |  | Cys40Arg, Ser112Asn, Asp119Glu, Ile237Val | Leu153Arg, Ser170Asn |  |
| PA202 | *exoU* | **8** | **320** |  |  |  |  |  |  |  |  |  |  |  |  | Cys40Arg, Ser112Asn, Asp119Glu, Ile237Val | Leu153Arg, Ser170Asn |  |
| PA217 | *exoU* | 1 | 1 |  |  |  |  |  |  |  |  |  |  |  |  | Cys40Arg, Ser112Asn, Asp119Glu, Ile237Val | Leu153Arg, Ser170Asn |  |
| PA219 | *exoU* | **≥5120** | **1280** |  |  |  |  |  |  |  |  |  |  |  |  | Cys40Arg, Ser112Asn, Asp119Glu, Ile237Val | Leu153Arg, Ser170Asn |  |
| PA220 | *exoU* | 0.5 | 0.5 |  |  |  |  |  |  |  |  |  |  |  |  | Cys40Arg, Ser112Asn, Asp119Glu, Ile237Val | Leu153Arg, Ser170Asn |  |
| PA221 | *exoU* | **2560** | **2560** |  |  |  |  |  |  |  |  |  |  |  |  | Cys40Arg, Ser112Asn, Asp119Glu, Ile237Val | Leu153Arg, Ser170Asn |  |
| PA233 | *exoU* | 0.5 | 0.5 |  |  |  |  |  |  |  |  |  |  |  |  | Asp161Gly, Asp207Asn, Ile237Val, Gly265Ser | Leu153Arg, Ser170Asn |  |
| PA17 | *exoS* | 0.25 | 0.25 |  |  |  |  |  |  |  |  |  |  |  |  | Asp161Gly, His182Gln |  |  |
| PA40 | *exoS* | 0.25 | 0.25 |  |  |  |  |  |  |  |  |  |  |  |  | Asp161Gly, His182Gln |  |  |
| PA149 | *exoS* | 0.25 | 0.25 |  |  |  |  |  |  |  |  |  |  |  |  | Ser112Asn, Asp119Glu, Ile237Val, Asn238Ser, Ala352Val |  |  |
| PA157 | *exoS* | 0.25 | 0.25 |  |  |  |  |  |  |  |  |  |  |  |  | Cys40Arg, Asp161Gly, His182Gln |  | Ala285Val |
| PA171 | *exoS* | 0.25 | 0.25 |  |  |  |  |  |  |  |  |  |  |  |  | Asp161Gly, Ile237Val |  |  |
| PA176 | *exoS* | 0.25 | 0.25 |  |  |  |  |  |  |  |  |  |  |  |  | Asp161Gly, His182Gln |  |  |
| PA181 | *exoS* | 0.25 | 0.25 |  |  |  |  |  |  |  |  |  |  |  |  | Gly157Asp, Asp161Gly, His182Gln |  |  |
| PA182 | *exoS* | 0.25 | 0.25 |  |  |  |  |  |  |  |  |  |  |  |  | Asp161Gly, His182Gln |  | Ser277Asn |
| PA188 | *exoS* | 0.5 | 32 |  |  |  |  |  |  |  |  |  |  |  |  | Asp161Gly, Gly319Val |  |  |
| PA189 | *exoS* | 0.25 | 16 |  |  |  |  |  |  |  |  |  |  |  |  | Asp161Gly, His182Gln, Gly319Val |  |  |
| PA193 | *exoS* | 0.25 | 0.25 |  |  |  |  |  |  |  |  |  |  |  |  | Asp161Gly, His182Gln | Leu153Arg, Ser170Asn |  |
| PA206 | *exoS* | 1 | 0.25 |  |  |  |  |  |  |  |  |  |  |  |  | Cys40Arg, Ala87val, Asp161Gly, His182Gln, Glu307Asp, Ile346Val | Thr135Ala, Leu153Arg | Ala115Glu |
| PA216 | *exoS* | 1 | 0.5 |  |  |  |  |  |  |  |  |  |  |  |  | Asp161Gly, His182Gln |  |  |
| PA218 | *exoS* | 0.5 | 0.5 |  |  |  |  |  |  |  |  |  |  |  |  | Asp161Gly, His182Gln | Leu153Arg, Ser170Asn |  |
| PA223 | *exoS* | 0.5 | 0.5 |  |  |  |  |  |  |  |  |  |  |  |  | Cys40Arg, Asp161Gly, His182Gln |  |  |
| PA224 | *exoS* | 0.25 | 0.25 |  |  |  |  |  |  |  |  |  |  |  |  | Asp161Gly, His182Gln |  |  |
| PA225 | *exoS* | 0.5 | 0.25 |  |  |  |  |  |  |  |  |  |  |  |  | Asp161Gly |  |  |
| PA227 | *exoS* | 0.5 | 0.25 |  |  |  |  |  |  |  |  |  |  |  |  | Asp161Gly, His182Gln |  |  |
| PA235 | *exoS* | 2 | 0.5 |  |  |  |  |  |  |  |  |  |  |  |  | Asp161Gly, His182Gln |  |  |

*, SNPs in *mexX, mexY, mexZ* and *oprM* are given in supplementary table 5 and 6. **Bold** numbers indicate resistance; No mutation in *fusA1* gene, all strains had Leu88Pro in ArmZ, so not shown in the table. Dark Box indicates presence of acquired resistance gene.
