# Supplementary material for: Differences in antimicrobial resistance between exoU and exoS isolates of Pseudomonas aeruginosa
Source: Eur J Clin Microbiol Infect Dis. 2025 Apr 22;44(7):1629–41. doi: 10.1007/s10096-025-05132-6 (PMC12241228; doi:10.1007/s10096-025-05132-6)
Supplement: Supplementary file 12 — Supplementary Material 12 [file 10096_2025_5132_MOESM12_ESM.docx]

Supplementary Table 4: Antimicrobial susceptibility of 20 *exoU* and 19 *exoS* lineages from keratitis that had whole genomes sequenced

| Antibiotics | *exoU*=20 | | *exoS*=19 | | P value |
| --- | --- | --- | --- | --- | --- |
|  | Susceptible  (%) | Resistant (%) | Susceptible  (%) | Resistant (%) |  |
| Ciprofloxacin | 25 | 75 | 42 | 58 | 0.32 |
| Levofloxacin | 45 | 55 | 90 | 11 | < 0.01 |
| Gentamicin | 45 | 55 | 100 | 0 | < 0.01 |
| Tobramycin | 45 | 55 | 90 | 11 | < 0.01 |
