# Supplementary material for: Differences in antimicrobial resistance between exoU and exoS isolates of Pseudomonas aeruginosa
Source: Eur J Clin Microbiol Infect Dis. 2025 Apr 22;44(7):1629–41. doi: 10.1007/s10096-025-05132-6 (PMC12241228; doi:10.1007/s10096-025-05132-6)
Supplement: Supplementary file 13 — Supplementary Material 13 [file 10096_2025_5132_MOESM13_ESM.docx]

Supplementary Table 5: Functional SNP changes in *gyrA, gyrB, parC, parE* and efflux pumps regulatory genes, leading to amino acid changes, and possession of *crpP* and *qnrVC1* of 20 *exoU* and 19 *exoS* keratitis isolates

| Strain ID | TTSS group | MICs of Antibiotics (µg/ml) | | DNA gyrase, DNA topoisomerase and efflux pump regulatory genes | | | | | | | | | Acquired resistance genes | |
| --- | --- | --- | --- | --- | --- | --- | --- | --- | --- | --- | --- | --- | --- | --- |
|  |  | Ciprofloxacin | Levofloxacin | *gyrA* | *parC* | *parE* | *mexR* | *nalC^1^* | *nalD* | *mexT^2^* | *mexS^3^* | *mexZ* | *crpP* | *qnrVC1* |
|  |  | Breakpoint ≤1,2, ≥ 4 µg/ml | Breakpoint  ≤2,4, ≥ 8 µg/ml |  |  |  |  |  |  |  |  |  |  |  |
| PA219 | *exoU* | **≥5120** | **640** | Thr83Ile | Ser87Ile | Asp533Glu | Val126Glu | Asp79Glu, Ser209Arg |  |  | Val73Ala | Gly89Ser |  |  |
| PA221 | *exoU* | **2560** | **2560** | Thr83Ile | Ser87Ile, Ala599Val |  | Val126Glu | Asp79Glu, Ser209Arg | Arg38Trp | Pro60Ser |  | Val43Gly |  |  |
| PA198 | *exoU* | **1280** | **320** | Thr83Ile | Ser87Ile | Asp533Glu | Val126Glu | Asp79Glu, Ser209Arg |  |  | Val73Ala | Gly89Ser |  |  |
| PA202 | *exoU* | **640** | **320** | Thr83Ile | Ser87Ile, Ala599Val | Asp533Glu | Val126Glu | Asp79Glu, Ser209Arg | Arg38Trp | Pro60Ser |  | Val43Gly |  |  |
| PA33 | *exoU* | **128** | **32** | Thr83Ile | Ser87Ile | Asp533Glu | Val126Glu | Asp79Glu, Ser209Arg |  |  | Val73Ala | Gly89Ser |  |  |
| PA217 | *exoU* | **64** | **32** | Thr83Ile | Ser87Ile | Asp533Glu | Val126Glu | Ser5Pro, Ser209Arg, Gly213Asp | Frameshift AA 160 to 213 | Pro60Ser |  |  |  |  |
| PA32 | *exoU* | **64** | **32** | Thr83Ile | Ser87Ile | Asp533Glu | Val126Glu | Asp79Glu, Ser209Arg |  |  | Val73Ala | Gly89Ser |  |  |
| PA35 | *exoU* | **64** | **32** | Thr83Ile | Ser87Ile | Asp533Glu | Val126Glu | Asp79Glu, Ser209Arg |  |  | Val73Ala | Gly89Ser |  |  |
| PA37 | *exoU* | **64** | **32** | Thr83Ile | Ser87Ile | Asp533Glu | Val126Glu | Asp79Glu, Ser209Arg |  |  | Val73Ala | Gly89Ser |  |  |
| PA82 | *exoU* | **64** | **4** | Thr83Ile | Ser87Ile | Asp533Glu | Val126Glu | Asp79Glu, Ser209Arg |  |  | Glu286Ala |  |  |  |
| PA31 | *exoU* | **32** | **32** | Thr83Ile | Ser87Ile | Asp533Glu | Val126Glu | Asp79Glu, Ser209Arg |  |  | Val73Ala | Gly89Ser |  |  |
| PA233 | *exoU* | **8** | 1 | Ser912Pro, Glu913Ala, Pro914Ala, Ala915Glu, Ala918Gly, Glu919Asn, Gly920Glu, Asn921Glu |  | Asp533Glu | Val126Glu | Ala145Val, Ser209Arg |  |  |  |  |  |  |
| PA169 | *exoU* | **2** | 0.25 |  |  | Asp533Glu | Val126Glu | Asp79Glu, Ser209Arg |  |  |  |  |  |  |
| PA220 | *exoU* | **2** | 0.25 | Thr83Ile | Ser87Ile | Glu459Val, Asp533Glu | Val126Glu | Asp79Glu, Ser209Arg | Ser32Asn |  |  |  |  |  |
| PA34 | *exoU* | **2** | 2 | Thr83Ile | Ser87Ile | Asp533Glu | Ala110Thr, Val126Glu | Asp79Glu, Ser209Arg |  | Pro60Ser |  |  |  |  |
| PA123 | *exoU* | 1 | 1 |  | Ser485Ala |  |  | Ser209Arg |  | Pro60Ser |  |  |  |  |
| PA127 | *exoU* | 1 | 0.25 |  |  |  |  | Ser209Arg |  | Pro60Ser |  |  |  |  |
| PA126 | *exoU* | 0.5 | 0.5 |  | Ser485Ala |  |  | Ser209Arg |  | Pro60Ser |  |  |  |  |
| PA162 | *exoU* | 0.5 | 0.5 | Ser912pro, Glu913Ala, Pro914Ala, Ala915Glu, Ala918Gly, Glu919Asn, Gly920Glu, Asn921Glu | Ala587Thr | Asp533Glu | Val126Glu | Ala145Val, Ser209Arg |  | Pro60Ser |  |  |  |  |
| PA175 | *exoU* | 0.25 | 0.25 | Ser912pro, Glu913Ala, Pro914Ala, Ala915Glu, Ala918Gly, Glu919Asn, Gly920Glu, Asn921Glu | Ser197Leu | Asp533Glu | Val126Glu | Ser209Arg |  |  |  | Asp83Glu, Leu138Arg |  |  |
| PA227 | *exoS* | **64** | **64** | Asp652Tyr |  |  |  | Ala186Thr |  | Pro60Ser |  |  |  |  |
| PA225 | *exoS* | **64** | **16** | Asp652Tyr |  |  |  | Glu153Asp, Ala186Thr |  | Pro60Ser |  |  |  |  |
| PA216 | *exoS* | **64** | 4 |  | Asp754Asn |  |  | Ala186Thr |  | Met7Val | Arg108Cys |  |  |  |
| PA223 | *exoS* | **64** | 1 | Ser912pro, Glu913Ala, Pro914Ala, Ala915Glu, Ala918Gly, Glu919As, Gly920Glu, Asn921Glu |  | Asp533Glu |  | Ser209Arg |  | Pro60Ser |  |  |  |  |
| PA224 | *exoS* | **16** | 1 | Ala659Tyr, Ser912pro, Glu913Ala, Pro914Ala, Ala915Glu, Ala918Gly, Glu919As, Gly920Glu, Asn921Glu |  |  | Val126Glu | Ser209Arg, Pro210Leu |  |  |  |  |  |  |
| PA235 | *exoS* | **16** | 0.5 |  |  | Ala473Val, Asp533Glu |  | Ser209Arg |  | Pro60Ser |  |  |  |  |
| PA218 | *exoS* | **8** | 1 |  |  |  | Val126Glu | Ser209Arg |  |  |  |  |  |  |
| PA171 | *exoS* | **4** | 2 |  |  |  |  | Ser209Arg | Thr188Ala | Met7Val, Pro60Ser |  |  |  |  |
| PA40 | *exoS* | **4** | 2 |  | Glu513Asp |  |  | Ser209Arg |  | Pro60Ser |  |  |  |  |
| PA17 | *exoS* | **2** | 1 |  |  | Asp533Glu |  |  | Thr11Asn | Pro60Ser |  |  |  |  |
| PA188 | *exoS* | **2** | 1 |  |  |  |  | Ser209Arg |  | Pro60Ser |  |  |  |  |
| PA206 | *exoS* | 1 | 0.5 | Ser912pro, Glu913Ala, Pro914Ala, Ala915Glu, Ala918Gly, Glu919Asn, Gly920Glu, Asn921Glu |  | Asp533Glu | Val126Glu | Ser209Arg |  | Glu26Gly, Pro60Ser | Lys17Thr, Ala75Val, Glu181Asp | Leu196Ile |  |  |
| PA193 | *exoS* | 1 | 0.25 |  |  |  | Val126Glu | Ser209Arg |  | Pro60Ser |  |  |  |  |
| PA181 | *exoS* | 1 | 0.25 |  |  |  |  |  |  | Pro60Ser | Ala75Val |  |  |  |
| PA182 | *exoS* | 1 | 0.25 |  |  |  | Val126Glu, Val132Ala | Ser209Arg |  | Met7Val |  |  |  |  |
| PA149 | *exoS* | 0.5 | 0.5 |  |  |  |  |  |  |  |  |  |  |  |
| PA176 | *exoS* | 0.5 | 0.25 |  |  |  |  | Ser209Arg |  | Pro60Ser |  |  |  |  |
| PA189 | *exoS* | 0.25 | 1 |  |  |  |  | Ser209Arg |  | Pro60Ser |  |  |  |  |
| PA157 | *exoS* | 0.25 | 0.5 |  |  |  |  |  |  | Pro60Ser |  |  |  |  |

**Bold** numbers indicate resistance; light blue font indicates new SNPs and red indicates new functional SNPs; No strain had mutations in GyrB and only strain PA157 had a mutation in NfxB (Arg82Leu) therefore these genes are not included in the table; ^1^, all strains had Gly71Glu in NalC*,* so not shown in the table; ^2^, all strains had deletion of AAs 81 and 82 followed by frameshift in MexT, so not shown in table; ^3^, all strains had Asp249Asn in MexS*,* so not shown in the table. Dark Box indicates presence of acquired resistance gene.
