# Supplementary material for: Differences in antimicrobial resistance between exoU and exoS isolates of Pseudomonas aeruginosa
Source: Eur J Clin Microbiol Infect Dis. 2025 Apr 22;44(7):1629–41. doi: 10.1007/s10096-025-05132-6 (PMC12241228; doi:10.1007/s10096-025-05132-6)
Supplement: Supplementary file 14 — Supplementary Material 14 [file 10096_2025_5132_MOESM14_ESM.docx]

Supplementary Table 7: The amino acid changes in fluoroquinolone-related resistance genes that were not significantly different between 20 *exoU* and 19 *exoS* keratitis isolates

| Gene | Amino acid changes |
| --- | --- |
| *gyrA* | Asp652Tyr, Ala659Tyr, Ser912Pro, Glu913Ala, Pro914Ala, Ala915Glu, Ala918Gly, Glu919Asn, Gly920Glu and Asn921Glu |
| *parC* | Ser197Leu, Ser485Ala, Glu513Asp, Ala587Thr, Ala599Val and Asp754Asn |
| *parE* | Ala473Val and Glu459Val |
| *mexA* | Lys76Gln, Lys289Arg and Asp373Glu |
| *mexC* | Ala31Val, Ala31Thr, Lys76Gln, Thr175Ala, Asp298Ala, His310Arg, Ser313Gly, Glu326Gly, Ala328Val, Ser330Ala, Glu347Lys, Ala361Pro, Val367Ala, Val370Ala, Ala378Thr, Pro383Ser and Ala384Val |
| *mexD* | Gln149His, Glu257Gln, Ser281Leu, Thr286Met, Thr304Ala, Val364Leu, Val434Ala, Ala536Ser, Ile610Val, Ala647Val, Glu650Gln, Asn669Asp, Ser685Gly, Val687Leu, Asn775Lys, Ser845Ala, Ser915Ala, Ala959Ser, Ile960Leu, Lys1031Arg and Ala1039Val |
| *mexE* | Asp353Glu, Asp370Glu and Pro397Gln |
| *mexF* | Asp230Ala, Ala598Glu and Asp606Glu |
| *mexR* | Ala110Thr and Val132Ala |
| *mexS* | Lys17Thr, Ala75Val, Arg108Cys, Glu181Asp, Asp249Asn and Glu286Ala |
| *mexT* | Met7Val, Glu26Gly, Pro60Ser and deletion of AAs 81 and 82 followed by frameshift |
| *mexX* | Leu12Pro, Thr15Ala, Asp25Glu, Lys26Glu, Pro28Ala, Glu29Asp, Glu31Gly, Ala33Thr, Asp35Glu, Lys46Arg, Val133Ile, Ser153Ala, Arg161Lys, Gln236Lys, Val331Leu, His338Arg, Gly344Asp, Gly384Asp and Val385Ala |
| *mexY* | Val240Met, Ala254Gly, Phe509Ser, Thr543Ala, Gly589Ala, Gln840Glu, Gly1035Asp, Asn1036Thr and Ile1040Thr |
| *mexZ* | Val43Gly, Asp83Glu, Leu138Arg, Leu196Ile and Asp249Asn |
| *nalC* | Ser5Pro, Gly71Glu, Ala145Val, Glu153Asp, Ala186Thr, Ser209Arg, Gly213Asp and Pro210Leu |
| *nalD* | Thr11Asn, Ser32Asn, Arg38Trp, Frameshift AA 160 to 213 and Thr188Ala |
| *oprJ* | Phe6Leu, Thr25Ile, Ala30Val, Gly120Ser, Ala163Thr, Gly184Ser, Ser264Asn and Asp351Ala |
| *oprM* | Val72Leu and Lys480Asn |
| *oprN* | Gly16Ser, Thr37Ala, Ala87Ser and Leu324Phe |

No strain had mutations in GyrB and only strain PA157 had a mutation in NfxB (Arg82Leu).
