# Supplementary material for: Differences in antimicrobial resistance between exoU and exoS isolates of Pseudomonas aeruginosa
Source: Eur J Clin Microbiol Infect Dis. 2025 Apr 22;44(7):1629–41. doi: 10.1007/s10096-025-05132-6 (PMC12241228; doi:10.1007/s10096-025-05132-6)
Supplement: Supplementary file 15 — Supplementary Material 15 [file 10096_2025_5132_MOESM15_ESM.docx]

Supplementary Table 10: Antimicrobial resistance differences between 132 Australian and 55 Indian keratitis isolates

| Total=187 | Australia (132) | India (55) | P value |
| --- | --- | --- | --- |
|  | Resistant (%) | Resistant (%) |  |
| Ciprofloxacin | 18.2 | 43.6 | < 0.01 |
| Levofloxacin | 12.9 | 27.3 | 0.03 |
| Gentamicin | 23.5 | 32.7 | 0.20 |
| Tobramycin | 13.6 | 29.1 | 0.02 |

Supplementary Table 11: Antimicrobial resistance differences between 20 Australian and 19 Indian keratitis isolates that had whole genomes sequenced

| Total=39 | Australia (20) | India (19) | P value |
| --- | --- | --- | --- |
|  | Resistant (%) | Resistant (%) |  |
| Ciprofloxacin | 50 | 84.2 | 0.04 |
| Levofloxacin | 10 | 57.9 | < 0.01 |
| Gentamicin | 0 | 57.9 | < 0.01 |
| Tobramycin | 5 | 63.2 | < 0.01 |

| Gene | Amino acid change | Frequency of possession (%)-*exoU:exoS* | Difference between *exoU* and *exoS* (p-value) | Correlation between amino acid change and resistance to ciprofloxacin (Spearman’s correlation coefficient R; p-value) | Correlation between amino acid change and resistance to levofloxacin (Spearman’s correlation coefficient R; p-value) |
| --- | --- | --- | --- | --- | --- |
| *gyrA* | Thr83Ile | 100:0 | < 0.01 | 0.64; < 0.01 | 0.79; < 0.01 |
| *parC* | Ser87Ile | 100:0 | < 0.01 | 0.64; < 0.01 | 0.79; < 0.01 |
| *parE* | Asp533Glu | 100:16.7 | < 0.01 | *0.33; 0.18* | 0.57; 0.01 |
| *mexR* | Val126Glu | 100:50 | 0.02 | *0.21; 0.39* | 0.51; 0.02 |
| *nalC* | Asp79Glu | 92.3:0 | < 0.01 | 0.57; 0.01 | 0.67; < 0.01 |
| *mexS* | Val73Ala | 53.8:0 | 0.04 | *0.33; 0.17* | 0.65; < 0.01 |
| *mexB* | Ser1041Glu | 69.2:0 | 0.01 | 0.51; 0.03 | 0.78; < 0.01 |
|  | Val1042Ala | 69.2:0 | 0.01 | 0.51; 0.03 | 0.78; < 0.01 |
| *mexC* | Glu251Gln | 53.8:0 | 0.04 | *0.33; 0.17* | 0.58; < 0.01 |
|  | **Ala262Glu** | 61.5:0 | 0.02 | *0.37; 0.12* | 0.65; < 0.01 |
|  | **Ala277Thr** | 53.8:0 | 0.04 | *0.33; 0.17* | 0.58; < 0.01 |
| *mexD* | Thr87Ser | 53.8:0 | 0.04 | *0.33; 0.17* | 0.58; < 0.01 |
| *oprJ* | Met69Val | 0:50 | 0.02 | *-0.21; 0.39* | *-0.27; 0.27* |
| *mexX* | Ala30Thr | 100:16.7 | < 0.01 | *0.39; 0.09* | *0.53; 0.18* |
| *mexY* | Ile536Val | 84.6:0 | < 0.01 | 0.50; 0.03 | *0.47; 0.05* |
| *crpP* | - | *92.3:50* | *0.07* | 0.48; 0.04 | *0.41; 0.08* |
| *qnrVC1* | - | *33.3:0* | *0.26* | *0.22; 0.36* | *0.39; 0.09* |

Supplementary Table 12: The significantly different amino acid changes resulting from differences in DNA sequences of DNA gyrase, DNA topoisomerase, efflux pumps and their regulatory genes, possession of acquired resistance genes between 13 *exoU* and 6 *exoS* Indian keratitis isolates, and correlations with resistance to fluoroquinolones

*Italicised* numbers indicate p > 0.05. Bold font indicates functional SNPs.

Supplementary Table 13: The significantly different amino acid changes resulting from differences in DNA sequences of efflux pumps and their regulatory genes, and acquired resistance genes of 13 *exoU* and 6 *exoS* Indian keratitis isolates

| Gene | Amino acid changes | Frequency of possession (%) – *exoU:exoS* | Difference between *exoU* and *exoS* (p-value) | Correlation between amino acid change and resistance to gentamicin (Spearman’s correlation coefficient R; p-value) | Correlation between amino acid change and resistance to tobramycin (Spearman’s correlation coefficient R; p-value) |
| --- | --- | --- | --- | --- | --- |
| *aph(3'')-Ib* | - | 92.3:0 | < 0.01 | 0.67; < 0.01 | 0.45; 0.04 |
| *aph(6)- Id* | - | 84.6:0 | < 0.01 | 0.57; < 0.01 | 0.57; < 0.01 |
| *armZ* | Cys40Arg | 100:16.7 | < 0.01 | 0.70; < 0.01 | 0.46; 0.04 |
|  | Ser112Asn | 100:0 | < 0.01 | 0.79; < 0.01 | 0.57; < 0.01 |
|  | Asp119Glu | 100:0 | < 0.01 | 0.79; < 0.01 | 0.57; < 0.01 |
|  | Asp161Gly | 0:100 | < 0.01 | -0.79; < 0.01 | -0.57; < 0.01 |
|  | His182Gln | 7.7:100 | < 0.01 | -0.57; < 0.01 | -0.57; < 0.01 |
|  | Ile237Val | 100:0 | < 0.01 | 0.79; < 0.01 | 0.57; < 0.01 |
| *mexX* | Ala30Thr | 100:16.7 | < 0.01 | 0.70; < 0.01 | 0.46; 0.04 |
| *mexY* | Ile536Val | 84.6:0 | < 0.01 | 0.78; < 0.01 | 0.78; < 0.01 |
|  | Gly589Ala | 61.5:0 | 0.02 | 0.73; < 0.01 | 0.73; < 0.01 |
|  | Gln840Glu | 61.5:0 | 0.02 | 0.73; < 0.01 | 0.73; < 0.01 |
|  | Asn1036Thr | 61.5:0 | 0.02 | 0.73; < 0.01 | 0.73; < 0.01 |

*Italicised* numbers indicate p > 0.05.

Supplementary Table 14. SNPs in the DNA mismatch repair (MMR) system genes in 13 *exoU* and 6 *exoS* Indian keratitis isolates

| Strains ID | TTSS group | *mutL* | *mutS* | *uvrD* | Total number of SNPs in *mut* genes^1^ | Total number of SNPs in AMR-associated genes^2^ |
| --- | --- | --- | --- | --- | --- | --- |
| PA31 | *exoU* | 1 | 0 | 4 | 5 | 54 |
| PA32 | *exoU* | 1 | 0 | 4 | 5 | 54 |
| PA33 | *exoU* | 1 | 0 | 4 | 5 | 54 |
| PA34 | *exoU* | 0 | 0 | 3 | 3 | 53 |
| PA35 | *exoU* | 1 | 0 | 4 | 5 | 54 |
| PA37 | *exoU* | 1 | 0 | 4 | 5 | 54 |
| PA82 | *exoU* | 0 | 1 | 5 | 6 | 43 |
| PA198 | *exoU* | 1 | 1 | 4 | 6 | 53 |
| PA202 | *exoU* | 1 | 1 | 5 | 7 | 51 |
| PA217 | *exoU* | 1 | 1 | 4 | 6 | 48 |
| PA219 | *exoU* | 1 | 1 | 4 | 6 | 54 |
| PA220 | *exoU* | 1 | 1 | 5 | 7 | 49 |
| PA221 | *exoU* | 1 | 1 | 5 | 7 | 51 |
| PA188 | *exoS* | 0 | 0 | 2 | 2 | 33 |
| PA189 | *exoS* | 0 | 0 | 2 | 2 | 33 |
| PA193 | *exoS* | 0 | 0 | 0 | 0 | 35 |
| PA206 | *exoS* | 0 | 1 | 0 | 1 | 79 |
| PA216 | *exoS* | 0 | 0 | 0 | 0 | 37 |
| PA218 | *exoS* | 0 | 0 | 0 | 0 | 31 |

^1,^ *mut* genes are *mutL, mutS* and *uvrD*; ^2,^ AMR-associated genes are *- gyrA, parC, parE, mexR, nalC, nalD, mexT, mexS, mexZ, mexA, mexB, oprM, mexC, mexD, oprJ, mexE, mexF, oprN, mexX, mexY, armZ, parR,* and *parS.*

Supplementary Table 15. Differences in SNPs possession in the DNA mismatch repair (MMR) system genes in 13 *exoU* and 6 *exoS* Indian keratitis isolates and association with SNPs in AMR-associated genes

| Gene | Frequency of SNP possession (%) – *exoU:exoS* | Difference between *exoU* and *exoS* (p-value) | Median SNP | | Interquartile Range (IQR) | | Correlation of MMR genes with total number of SNPs in AMR-associated genes^1^ (R, p value) |
| --- | --- | --- | --- | --- | --- | --- | --- |
|  |  |  | *exoU* | *exoS* | *exoU* | *exoS* |  |
| *mutL* | 84.6:0 | p < 0.01 | 1 | 0 | 0 | 0 | R = 0.57; p = 0.01 |
| *mutS* | 53.9:16.7 | P = 0.18 | 1 | 0 | 1-0 | 0 | R = 0.13; p = 0.59 |
| *uvrD* | 100:3.3 | p < 0.01 | 4 | 0 | 5-4 | 2-0 | R = 0.31; p = 0.20 |

^1,^ AMR-associated genes are *- gyrA, parC, parE, mexR, nalC, nalD, mexT, mexS, mexZ, mexA, mexB, oprM, mexC, mexD, oprJ, mexE, mexF, oprN, mexX, mexY, armZ, parR,* and *parS.*
